# Alignment-free genome comparison enables accurate geographic sourcing of white oak DNA

**DOI:** 10.1186/s12864-018-5253-1

**Published:** 2018-12-10

**Authors:** Kujin Tang, Jie Ren, Richard Cronn, David L. Erickson, Brook G. Milligan, Meaghan Parker-Forney, John L. Spouge, Fengzhu Sun

**Affiliations:** 1 Quantitative and Computational Biology Program, University of Southern California, Los Angeles, CA 90089 USA; 2 Pacific Northwest Research Station, USDA Forest Service, Corvallis, OR 97331 USA; 3 DNA4 Technologies LLC, bwtech@UMBC Research & Technology Park, Baltimore, MD 21227 USA; 4 Conservation Genomics Laboratory, Department of Biology, New Mexico State University, Las Cruces, NM 88003 USA; 5 World Resources Institute, Washington, DC 20002 USA; 6 National Center for Biotechnology Information, National Library of Medicine, National Institutes of Health, Bethesda, MD 20894 USA; 7 Centre for Computational Systems Biology, School of Mathematical Sciences, Fudan University, Shanghai, 200433 China

## Abstract

**Background:**

The application of genomic data and bioinformatics for the identification of restricted or illegally-sourced natural products is urgently needed. The taxonomic identity and geographic provenance of raw and processed materials have implications in sustainable-use commercial practices, and relevance to the enforcement of laws that regulate or restrict illegally harvested materials, such as timber. Improvements in genomics make it possible to capture and sequence partial-to-complete genomes from challenging tissues, such as wood and wood products.

**Results:**

In this paper, we report the success of an alignment-free genome comparison method, ${d}_2^{\ast },$ that differentiates different geographic sources of white oak (*Quercus*) species with a high level of accuracy with very small amount of genomic data. The method is robust to sequencing errors, different sequencing laboratories and sequencing platforms.

**Conclusions:**

This method offers an approach based on genome-scale data, rather than panels of pre-selected markers for specific taxa. The method provides a generalizable platform for the identification and sourcing of materials using a unified next generation sequencing and analysis framework.

**Electronic supplementary material:**

The online version of this article (10.1186/s12864-018-5253-1) contains supplementary material, which is available to authorized users.

## Background

The annual trade in natural resources represents $3.35 T of global imports and $3.25 T of global exports [1], and it supports the world’s supply of food, building materials, and fiber. Disturbingly, Global Financial Integrity estimates the annual, transnational illegal trade in natural resources to be valued at $90B-$276B [2]. Illegal trade in forest products is the single largest component of illegal trade, accounting for ~ 50% of estimated annual losses. Illegal logging contributes significantly to deforestation and forest degradation, and these have cascading impacts on natural resource conservation, global biodiversity, climate change mitigation, and the economic health of billions of people [3]. To mitigate trafficking of illegally-sourced wood, the United States (2008), European Union (2010) and Australia (2012) adopted regulations that prohibit the import, export, transport, purchase or sale of illegally harvested timber and plant products. These regulations can impose civil and criminal penalties on buyers and suppliers of wood products who fail to adopt “due care” controls. A key component of due care is that wood or wood products entering or exiting the U.S. must declare the scientific name and geographic source of the wood. Despite this requirement, mislabeling and document falsification are widespread because few methods are available to validate these declarations [4].

Historically, verification of wood has been accomplished using features such as density, scent, cellular composition, and vessel distribution [5]. This approach is rapid, but generally incapable of identifying trees to species or predicting their geographic origin [6, 7]. Chemical [8, 9] and genetic [10, 11] approaches are increasingly used to provide more accurate species identifications [4], but determining geographic origin continues to be a daunting task [12–15]. Here, we demonstrate an efficient use of next generation sequencing (NGS) data to predict the geographic source of white oak species (*Quercus* subg*. Quercus*). Unlike traditional genetic analysis, our approach uses whole genome DNA sequence data without a priori selection of marker loci. This work extends studies showing that background-adjusted alignment-free sequence comparison measures (CVTree [16]; ${d}_2^{\ast }$ and ${d}_2^s$[17–20]) offer improvements over other comparison measures (Euclidean, Manhattan, *d*_2_ distances [21–23]) for the comparison of molecular sequences. We chose white oaks for this analysis for three reasons: white oaks include hardwood species with the highest export volume from the U.S. [24]; they cannot be readily discriminated using wood anatomy, and at least one species (*Q. mongolica* from Russia) is protected by the Convention on International Trade in Endangered Species of Wild Fauna and Flora, and was the focus of a U.S. Lacey Act conviction [25].

Using NGS data from 92 white oaks from North America (NA), Europe (EU), and Asia (AS), we show that for each sample the two most similar white oak trees according to the ${d}_2^{\ast }$ dissimilarity measure are from the same geographic provenance based on small sequencing quantities (e.g., 50 Mbp). Finally, we show that K-nearest neighbors (KNN) classification yields close to 100% classification accuracy of geographic provenance, even with data generated from different sequencing platforms and genome reduction methods. Our study demonstrates that continental origin of trees can be accurately predicted using KNN coupled with ${d}_2^{\ast }$ dissimilarity, and that the method offers a simple and unified approach for geographic and taxonomic identification that can be applied to any biological sample.

## Results

### Genomic dissimilarity analyses based on ${d}_2^{\ast }$ resolve oak geographic origins

**Fig. 1:**
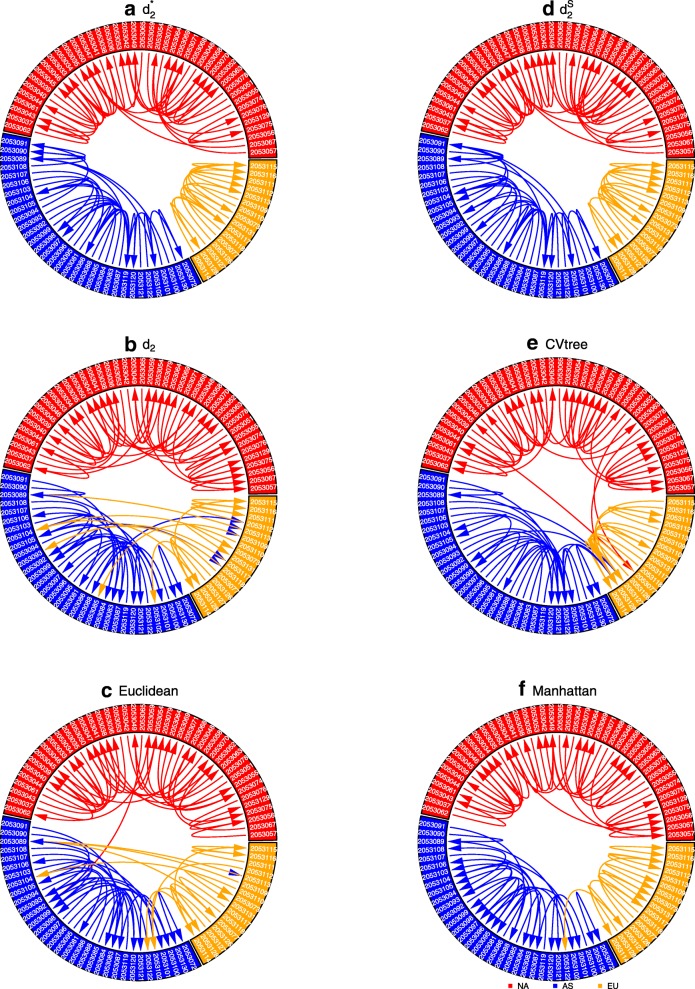
The circular plots of 92 white oak tree samples based on the six dissimilarity measures: **a**) $$ {d}_2^{\ast } $$, **b**)$$ {d}_2^s $$ *d*_2_, **c**) Euclidean, **d**) $$ {d}_2^s $$, **e**) CVTree and (**f**) Manhattan, using 100 Mbp of next generation sequencing data. Different sectors correspond to different continents, with NA in red, EU in orange, and AS in blue; GenBank accession numbers are identified in white font. Within each sector, samples are sorted by their longitude, so that samples that are geographically close are also close to each other in the figure. The most similar tree sample to each sample is linked. The *k*-mer length is 12 and the Markov order of the background sequence is 10 for $$ {d}_2^{\ast } $$, $$ {d}_2^s, $$ and CVTree. The most similar sample to each sample according to $$ {d}_2^{\ast } $$ and $$ {d}_2^s $$ are from the same continent-of-origin

We used six alignment-free distance/dissimilarity measures (Manhattan, Euclid, *d*_2_ [26], CVTree [16], ${d}_2^{\ast }$ and ${d}_2^s$ [17–19]) based on the relative frequencies of k-mers to calculate pairwise distances of white oak tree samples based on DNA samples of 50, 100 and 300 Mbp. Figure 1 shows the circular plots [27] of the oak trees at sequencing quantity of 100 Mbp using the six dissimilarity measures (circular plots at sequencing quantities of 50 Mbp and 300 Mbp are shown as Additional File 1 (Figure S2). In each plot, the most similar sample to each of the reference specimens is linked. Of the six dissimilarity measures, only ${d}_2^{\ast }$ and ${d}_2^s$ showed 100% accuracy in linking a sample to its continent-of-origin.

**Fig. 2:**
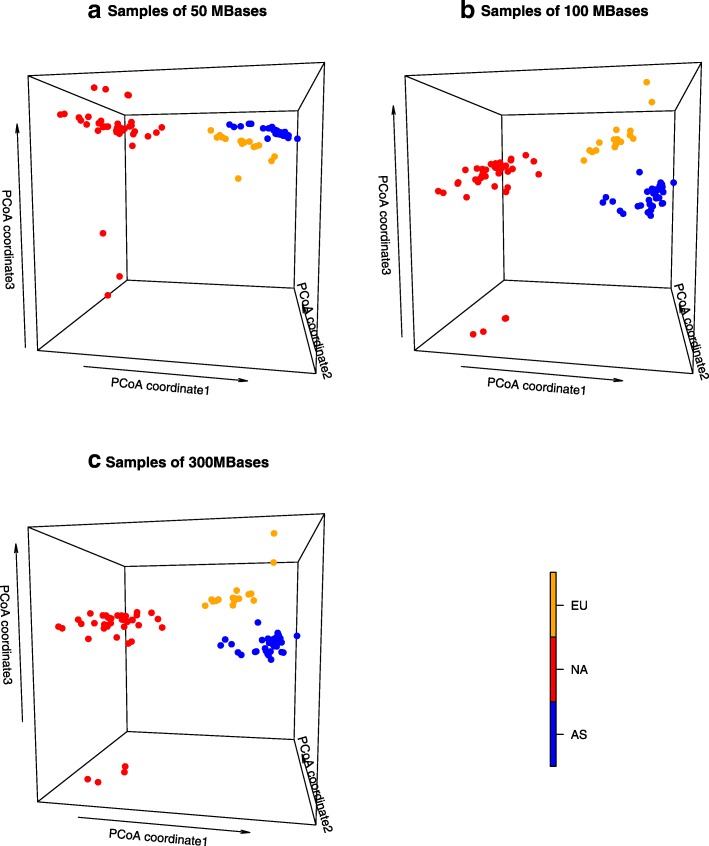
The principal coordinate plots (PCoA) of 92 white oak tree samples based on the ${d}_2^{\ast }$ dissimilarity values using **a**) 50 Mbp, **b**) 100 Mbp, and **c**) 300 Mbp of next generation sequencing data. The *k*-mer length is 12 and the Markov order of the background sequence is 10. With sequence quantity of 100 and 300 Mbp, the Europe (EU) and Asia (AS) samples are clearly separated in the PCoA plots. Four western North America samples separate from the other eastern North America samples

Principal coordinate analysis of the pairwise ${d}_2^{\ast }$dissimilarities among 92 samples at sequencing quantities of 50, 100 and 300 Mbp showed that the first three principal coordinates accounted for ~ 25% of variance in dissimilarities. Samples could be separated into three distinct groups corresponding to their continental origins (Fig. 2) using the first three principal coordinates. The first principal coordinate separates all samples from different primary continents, i.e., North America and Europe/Asia, and the third principal coordinate separates samples from Europe (EU) versus Asia (AS). Although the second principal coordinate of the AS tree samples is generally larger than that of the EU tree samples, it does not completely separate the EU tree samples from the AS tree samples. The latitudes of the tree samples are not strongly associated with the first three principal coordinates (Additional File 2, Figure S3).

### Mean ${\boldsymbol{d}}_{\mathbf{2}}^{\ast }$ is smaller within continents than among continents

**Fig. 3:**
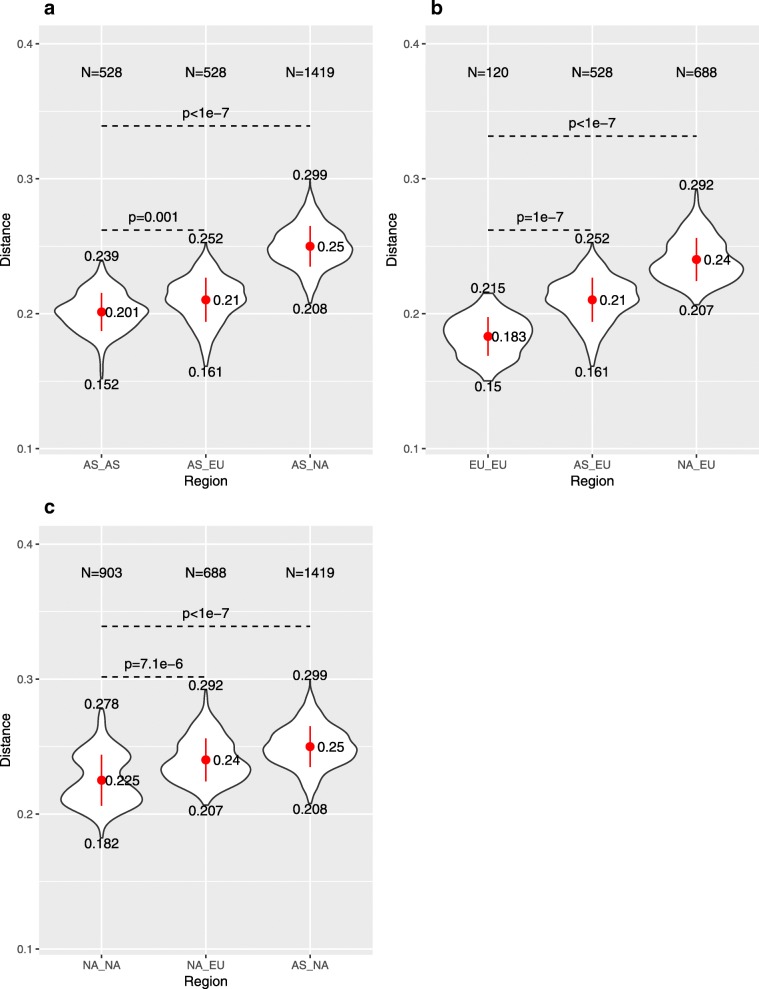
Comparison of intra- and inter-continental ${d}_2^{\ast }$ dissimilarities with sequence quantity of 100 Mbp based on **a**) Asian, **b**) European, and **c**) North American sources. The *k*-mer length is 12 and the Markov order of the background sequence is 10. The *p*-values were calculated based on the Wilkinson-Man-Whitney test statistic and by permuting the continental labels of the white oak tree samples 10^7^ times. The inter-continental ${d}_2^{\ast }$ dissimilarities are significantly higher than intra-continental ${d}_2^{\ast }$ dissimilarities

The distributions of ${d}_2^{\ast }$ dissimilarity values were compared for white oaks within- and among-continents across all samples, and at three sequencing quantities (100 Mbp in Fig. 3; 50 and 300 Mbp in Additional File 3, Figure S4). Mean pairwise dissimilarities within a continent are significantly smaller than dissimilarities among different continents (Wilcoxon-Mann-Whitney test; *p* < 0.001). Within continents, white oak samples from EU show the highest similarity, followed by white oaks from AS; white oaks from NA showed the greatest average within-continent divergence. Among-continent comparisons showed that EU and AS have the highest similarity, and that white oaks from NA are almost equally dissimilar to EU and AS white oaks; these dissimilarities mirror the chloroplast genome-based phylogenic estimates for these same taxa [28]. Our observations suggest that the continental origin of white oak samples can be predicted by KNN, in which the continent-of-origin for a sample is predicted as the continent containing the closest neighbors based on ${d}_2^{\ast }$.

**Fig. 4:**
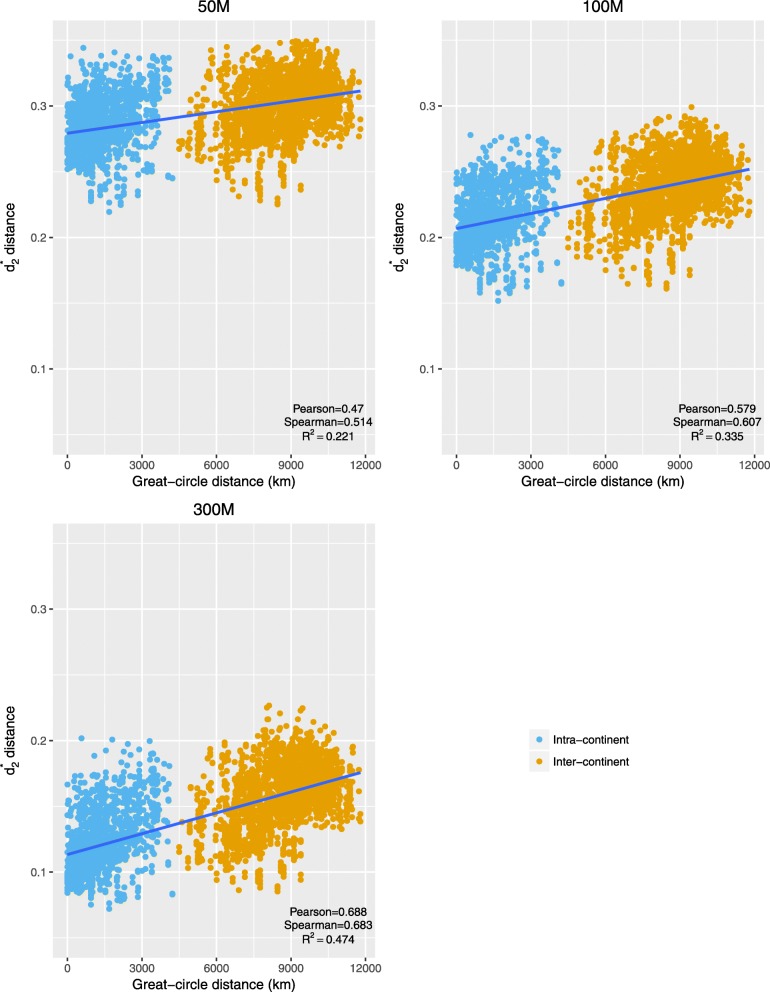
The relationship between ${d}_2^{\ast }$ dissimilarity values and great-circle distances among the tree samples based on sequence quantity of 50, 100 and 300 Mbp. The ${d}_2^{\ast }$ dissimilarity values are significantly positively associated with the great-circle distances and both the Pearson and Spearman correlation coefficients increased with sequence quantity. However, the ${d}_2^{\ast }$ dissimilarity is not a strong predictor of great-circle distance

Figure 4 shows the relationship between ${d}_2^{\ast }$ dissimilarity and the great-circle distance of oak samples. The ${d}_2^{\ast }$ dissimilarity and the great-circle distance are significantly and positively associated, and correlation coefficients between these measures increase with increasing quantity of DNA sequence. For example, at a sequencing quantity of 50 Mbp, 22.1% of the ${d}_2^{\ast }$ variation can be explained by the great-circle distance (Pearson *R =* 0.470). By increasing the sequencing quantity to 300 Mbp, 47.4% of the${d}_2^{\ast }$ variation can be explained by the great-circle distance (Pearson *R* = 0.688). Despite the statistically significant associations between great-circle distance and ${d}_2^{\ast }$, individual variation in pairwise ${d}_2^{\ast }$ is sufficiently high that predicted pairwise great-circle distances are of limited practical value.

### KNN predictions are robust to multiple sources of errors

Based on the ${d}_2^{\ast }$dissimilarity measure, we used KNN to build a predictive model for the continental origin of tree samples. Table 1 shows the mean prediction accuracy using KNN over 100 training and test data sets for different sequencing quantities, values of K, and sizes of training data. In all cases, K = 1 and K = 2 have similarly high prediction accuracies. The prediction accuracy of KNN increases with sequencing quantity and the size of training samples. For example, when *K* = 1 and training size is at least 75 reference samples, KNN prediction accuracy can reach 100%, even when the quantity of sequence is as low as 50 Mbp. With only 15 reference samples, KNN prediction accuracy ranges from 89% (50 Mbp) to 96% (300 Mbp).

**Table 1:** KNN accuracy on test data for different sample sizes, test sizes, training sizes and different numbers of neighbors K used

Test size	Training size	K = 1	K = 2	K = 3	K = 4	K = 5	K = 6	K = 7	K = 8	K = 9	K = 10
Samples of 50 MBases											
1	91	1.00	1.00	1.00	1.00	1.00	1.00	0.93	0.97	0.88	0.94
17	75	1.00	1.00	0.99	0.99	0.97	0.97	0.95	0.96	0.94	0.96
32	60	0.99	0.99	0.97	0.97	0.94	0.95	0.93	0.95	0.93	0.95
47	45	0.98	0.98	0.95	0.96	0.93	0.94	0.93	0.95	0.91	0.93
62	30	0.95	0.95	0.92	0.93	0.90	0.93	0.90	0.92	0.89	0.91
77	15	0.89	0.89	0.85	0.87	0.81	0.82	0.79	0.77	0.73	0.67
Samples of 100 MBases											
1	91	1.00	1.00	1.00	1.00	1.00	1.00	0.98	1.00	0.96	1.00
17	75	1.00	1.00	0.99	0.99	0.98	0.98	0.97	0.99	0.97	0.99
32	60	1.00	1.00	0.98	0.99	0.97	0.98	0.96	0.97	0.95	0.96
47	45	0.99	0.99	0.97	0.98	0.96	0.97	0.95	0.97	0.95	0.97
62	30	0.98	0.98	0.95	0.96	0.94	0.96	0.93	0.95	0.90	0.91
77	15	0.93	0.93	0.90	0.91	0.86	0.84	0.81	0.78	0.70	0.67
Samples of 300 MBases											
1	91	1.00	1.00	1.00	1.00	1.00	1.00	1.00	1.00	1.00	1.00
17	75	1.00	1.00	1.00	1.00	1.00	1.00	0.99	1.00	1.00	1.00
32	60	1.00	1.00	0.99	1.00	0.99	1.00	0.98	0.99	0.98	0.99
47	45	1.00	1.00	0.99	0.99	0.98	0.99	0.98	0.99	0.97	0.98
62	30	0.99	0.99	0.97	0.98	0.96	0.97	0.94	0.95	0.91	0.92
77	15	0.96	0.96	0.92	0.93	0.86	0.86	0.81	0.79	0.74	0.71

Table 2 shows the average prediction accuracy of KNN with 5% additional simulated sequencing error in test data for different sequence quantities, values of K, and sizes of training data. While prediction accuracy decreases with increasing sequencing error rate, prediction accuracies can reach 100% at sequencing quantity of 100 Mbp and a training set of 91 samples. At sequencing quantity of 300 Mbp, the prediction accuracy can reach 100% with a training set of 75. For most modern sequencers, the per-position sequencing error rate is much lower than 5%; for example, the sequencing error rate for Illumina is about 0.1% [29]. Our results show that KNN can predict the continental origins of oaks based on ${d}_2^{\ast }$ dissimilarity values at a high accuracy, and the prediction accuracy is robust to sequencing errors if the sequencing quantity in the training data set is at least 100 Mbp.

**Table 2:** KNN accuracy on test data with 5% simulated sequencing error for different sample sizes, test sizes, training sizes and different numbers of neighbors

Test size	Training size	K = 1	K = 2	K = 3	K = 4	K = 5	K = 6	K = 7	K = 8	K = 9	K = 10
Samples of 50 MBases											
1	91	0.93	0.93	0.90	0.92	0.90	0.91	0.84	0.85	0.71	0.81
17	75	0.88	0.88	0.84	0.87	0.82	0.83	0.76	0.79	0.71	0.78
32	60	0.86	0.86	0.80	0.83	0.78	0.79	0.74	0.79	0.75	0.82
47	45	0.80	0.80	0.73	0.76	0.69	0.74	0.71	0.76	0.73	0.80
62	30	0.77	0.77	0.68	0.75	0.71	0.77	0.74	0.79	0.78	0.81
77	15	0.66	0.66	0.64	0.68	0.70	0.74	0.74	0.73	0.69	0.67
Samples of 100 MBases											
1	91	1.00	1.00	0.98	1.00	1.00	1.00	0.99	0.99	0.82	0.92
17	75	0.99	0.99	0.96	0.98	0.93	0.93	0.86	0.90	0.82	0.88
32	60	0.96	0.96	0.92	0.94	0.87	0.89	0.84	0.87	0.83	0.88
47	45	0.93	0.93	0.87	0.90	0.83	0.87	0.83	0.88	0.83	0.88
62	30	0.86	0.86	0.79	0.84	0.78	0.83	0.80	0.84	0.82	0.85
77	15	0.77	0.77	0.72	0.76	0.73	0.75	0.73	0.72	0.68	0.65
Samples of 300 MBases											
1	91	1.00	1.00	1.00	1.00	1.00	1.00	0.98	0.99	0.95	0.98
17	75	1.00	1.00	1.00	1.00	0.98	0.99	0.95	0.97	0.93	0.95
32	60	0.99	0.99	0.97	0.98	0.94	0.95	0.92	0.95	0.93	0.95
47	45	0.98	0.98	0.94	0.95	0.92	0.93	0.91	0.94	0.92	0.95
62	30	0.95	0.95	0.90	0.93	0.90	0.93	0.89	0.91	0.87	0.89
77	15	0.88	0.88	0.84	0.86	0.81	0.82	0.78	0.76	0.72	0.70

### KNN predictions are robust to sequencing technologies

**Fig. 5:**
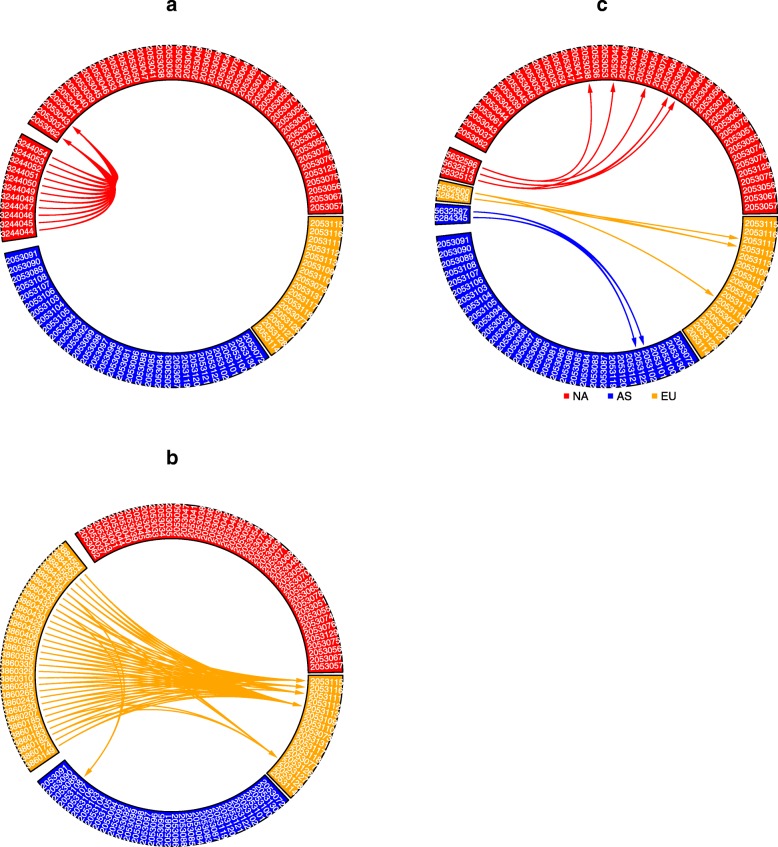
The circular plots for independent samples sequenced using (**a**) Illumina NGS of a California Valley Oak tree, (**b**) a mixture of short- and long- read from with both Illumina and PacBio sequencing of the Pendunculate Oak tree, and (**c**) seven diverse tree samples using RAD-seq. The ${d}_2^{\ast }$ dissimilarity measures of each independent sample with the 92 reference samples were calculated and the two most similar reference samples are linked

We next applied the KNN approach to predict the continental origins of white oak samples from independent laboratories using a) different Illumina sequencing platforms, b) various short- and long- reads sequencing technologies, and c) RAD sequencing. The results are summarized in Fig. 5. The corresponding figures using ${d}_2^s$ and Manhattan dissimilarity measures are shown in Additional File 4 (Additional file 4: Figure S5). The first example is a white oak sample from NCBI using Illumina NGS data produced by independent laboratories (described in Methods). For all 11 NGS data sets from the Californian Valley Oak genome project, the most similar sequence in our training set was a tree from the same species (*Quercus lobata*; SRR2053043), also from California. For these libraries, the second most similar sequence from our training set came from a phylogenetically closely-related species from a proximal geographic region in western North America (*Q. garryana*, SRR2053062; Oregon, USA) [28]. For all the 11 data sets, the top 20 most closely related samples were all from NA. Therefore, KNN with *K* = 1 to 20 can accurately predict the continental origins of the tree, irrespective of the library preparation methods and Illumina sequencing technologies with different read lengths (e.g., 100 bp single-end vs. 150 bp paired-end).

The second example is a white oak sample from NCBI that was sequenced using a mix of short- and long-read sequencing technologies (described in Methods). For the eight Illumina data sets from the Pendunculate Oak genome project (*Q. robur;* provenance near Lausanne, Switzerland), the smallest ${d}_2^{\ast }$ dissimilarity between each data set and the 92 training samples ranged from 0.23–0.24. The two most similar sequences in our training set were from a closely related European species (*Q. petraea*; SRR2053113, SRR2053111) of German provenance. For these libraries, the five most similar samples included *Q. robur* and *Q. petraea*, all from EU. Therefore, we can make accurate predictions for continent-of-origin based on KNN with *K* = 1 to 5. This genome project also used long-read PacBio sequencing [30], a method that shows a higher per-position error rate of 11–15%. The smallest ${d}_2^{\ast }$ dissimilarity values between the Pendunculate Oak genome PacBio sequence and the training data were 0.42, indicating substantial differences attributable to the different sequencing platforms. Despite these large dissimilarities, the most similar training samples still included *Q. robur* and *Q. petraea* from EU. Therefore, if we use 1NN as the classifier using ${d}_2^{\ast }$ dissimilarities, the prediction accuracy is 100% irrespective of the sequencing platform used. Only in two cases, the second most similar samples are from AS.

Genome-reduction methods are increasingly used in population genomic analysis, so we tested KNN classification for continent-of-origin using seven white oak samples sequenced with the RAD-Seq technique [31], including 2 from AS, 2 from EU, and 3 from NA [32]. These samples were highly divergent from our 92 white oak reference samples, with ${d}_2^{\ast }$ dissimilarities ranging between 0.483 and 0.491. In two cases, identical DNAs were compared by RAD-Seq and shotgun sequencing: these include *Q. mongolica* (${d}_2^{\ast }$ = 0.488) and *Q. petraea* (${d}_2^{\ast }$ = 0.485). Overall, we found that the top two highest similarity comparisons for all RAD-Seq samples were reference sequences from the correct continental origin, indicating 100% prediction accuracy. For the identical DNAs sampled by two sequencing methods, RAD-Seq samples did not show minimum ${d}_2^{\ast }$ dissimilarity with their corresponding shotgun Illumina samples, but were instead ranked 5th (*Q. mongolica*) and 6th (*Q. petraea*) among the 92 pairwise comparisons. These results indicate that KNN classification of ${d}_2^{\ast }$ dissimilarities lack the specificity required for individual identification for samples obtained using different genome sampling methodologies, but that geographic prediction accuracy is sufficiently high for continent-of-origin prediction in white oaks.

### Assigning confidence to the predicted continental origins

The predicted continent of origin of a wood sample depends on the reference samples and the NGS data. To evaluate the influence of reference sample composition on prediction accuracy, we defined reference-confidence (RC) by random sampling of the references and NGS-confidence (NC) by random sampling of reads (see Methods section for details). We calculated the RC and the NC of the predicted continental origins of the California Valley Oak tree and the Swiss Pendunculate Oak tree. For the 11 NGS data sets derived from the California Valley Oake, all the RC and NC indicate 100% accuracy for K = 1 to 10. Among the 30 data sets derived from the Swiss Pendunculate Oak, 14 data sets have RC of 100% and 13 data sets have RC value between 99 and 100%. Only three data sets, SRR3860432, SRR3860434, SRR3860435, have RC value of 66, 90, and 74%, respectively. For all these data sets, they were predicted to come from EU/AS with 100% confidence. In terms of prediction variation due to NGS reads data, among the 207 non-overlapping data sets from the 30 reads sets, the predicted continental origins for 205 sets were EU and 2 data sets with predicted origin as AS. Therefore, the predictions were not affected by the different non-overlapping data sets of DNA sequences.

## Discussion

In this paper, we show that the continental origin of plant materials can be identified based on the application of ${d}_2^{\ast }$ and KNN classification from a small sample of random DNA sequences. The challenge of identifying geographic provenance and species identification is not limited to illegal timber trade, but is problematic for all biological materials. Plant material identification has applications to food safety [33–35] and product labeling [36], and is of increasing importance to conservation and sustainable agriculture [15]. Introductions of exotic invasive species can occur as a result of mislabeling in the horticulture trade [37]. Game meat sold commercially in the U.S. and elsewhere is often mislabeled [38, 39]. Genetic approaches are used to identify illegal products in trade [4, 40], but as traditionally applied, they can be expensive, time-consuming to develop, and difficult to scale. The method we describe here can be applied uniformly to all biological materials, eliminating the need for pre-defined panels of genetic markers. The approach is robust to different laboratory methods and sequencing environments, making it easy to automate for speed and consistency. This advance represents a major step forward in determining geographic provenance and taxonomic identity through genome-scale comparisons, and this is an essential prerequisite in order for genomics technologies to make an impact on this daunting global challenge. We recognize that the sourcing of wood may be particularly challenging due to the difficulties of recovering sufficient DNA from wood tissues. However, ongoing work suggests this issue is superable, even for challenging woods like dense and pigmented rosewood species [41]. Therefore, joint development of laboratory methods and bioinformatic tools like those described here maybe especially successful.

The use of genetic information to identify the source of natural products is a practical application of population and landscape genetics that is widely applied to track the trade of protected species [40]. The advantages of genomics approaches include: (1) identification and determination of geographic origin are based upon a century of established population genetics and evolutionary theory because the data are explicitly genetic; (2) large reference databases may be constructed rapidly by leveraging pre-existing herbarium or museum collections and using only milligrams to tens of milligrams from a diversity of tissues; and (3) the sensitivity of analysis is easily scalable by increasing sequencing depth. For the alignment-free methods discussed here, there is no need to develop specific genetic markers, which greatly simplifies the process. Finally, the method can be applied to *any* biological material without a priori knowledge of the species’ identity or geographic source. In contrast, the traditional population genetic methods (e.g., species-specific SNP arrays) may offer comparable resolution or cost efficiencies on a per-sample basis once such genetic methods are developed, but they require much more development efforts and much greater a priori knowledge; consequently, their practical utility for addressing such challenges as identifying illegally sourced natural products has been severely limited. The potential for universal procedures makes genomics approaches especially promising for highly processed materials, such as mixtures, composites, or veneers that many include multiple unrelated biological materials.

Chemical methods, such as DART-TOF mass spectrometry, have some similarities as a universal approach to sample identification. Data are captured in a uniform fashion from diverse sample types, and then compared to a reference database of known materials for identification [13, 42]. DART-TOF mass spectrometry can identify and categorize novel samples based upon their chemical profiles, and has the potential to differentiate populations [13]. However, it is unclear whether the precision of the chemical analysis methods is equivalent to that of genomics or genetic marker analysis methods. Further, unlike genetics, there is no theory that enables extrapolation from one chemical profile to another, so accurate chemical identification will be entirely determined by the scale of available reference databases. Stable isotopes have also been used to identify the geographic source of natural products [43]. Like chemical analysis, analysis of stable isotopes is entirely based upon availability of relevant empirical databases; there is no established theory linking geographic variation in mineral isotopes and that in co-occurring biological samples. While isotopes are considered to provide location information independent of population genetic structure and even species identity, isotopic fractionation can in fact depend on these factors. Presently, stable isotope analysis may be effective for determining geographic origin among sites dispersed by at least hundreds of kilometers; however, they lack the precision to differentiate more finely and, unlike genetic information, cannot differentiate individuals. Future work that directly compares and contrasts these approaches across a wide variety of different sets of common samples is essential, as a combination of approaches may ultimately be needed for authoritative assessment of taxonomic identity and geographic origin.

We evaluated six alignment-free sequence comparison dissimilarity measures for predicting continent-of-origin based on NGS short read data from 92 white oak trees sampled in North America, Europe, and Asia. The recently developed background-adjusted dissimilarity measures ${d}_2^{\ast }$ and ${d}_2^s\kern0.5em$correctly predicted the continent-of-origin with highest accuracy, and we explored prediction accuracies of ${d}_2^{\ast }$–based KNN classification for the continental origins of white oak samples. We found that prediction accuracy reaches 100% with as little as 50 Mbp sequence data (< 1/10 the size of the white oak genome), small values of *K* (1–2), and a modest training database of 75 samples. With a larger training database of 92 trees, the prediction accuracy is 100% for 100 Mbp of sequence data and larger values of K (< 6). Although the prediction accuracy of KNN decreases with increasing sequencing error, the prediction accuracy of KNN can be as high as 100% with 5% additional errors over the observed experimental errors, as long as the sequencing quantity is at least 100 Mbp. This suggests that ${d}_2^{\ast }$-based classification is sufficiently accurate for portable nanopore-based sequencers [44]. This would expand the utility of field-based DNA sequencing beyond simple organisms with small genomes to organisms with larger genomes, and open new applications for remote field-based studies that put DNA-based identification closer to supply regions with the greatest risk of illegal harvesting.

To evaluate the applicability of ${d}_2^{\ast }$–based KNN prediction of continent-of-origin for oak DNA sequence data from different library preparation methods and different sequencing platforms, we predicted the continental origins of tree genome sequences obtained from NCBI that were based on whole genome sequencing (*Q. lobata* from NA; *Q. robur* from EU) and one genome reduction technique (RAD-Seq; seven trees from AS, EU and NA). We found that different library preparation methods and laboratories had the smallest impact on ${d}_2^{\ast }$ dissimilarity, that different sequencing platforms (Illumina versus PacBio) had a larger effect, and that different genome sampling methods (shotgun sampling versus RAD-Seq) had the largest effect. Surprisingly, KNN still predicted continental origins of oak trees perfectly for all of these methodological permutations (laboratory; sequencer; genome sampling), as long as the query tree sample NGS data was compared with reference tree data derived from a single, accurate sequencing platform (Illumina, in our case). Although technical and sampling errors in data acquired using PacBio or RAD-Seq are larger than those in the reference data sets generated using Illumina sequencing, data from these alternative methods still show smaller dissimilarities to the correct geographic assignment, and this allows KNN to accurately predict the continental origins of tree samples.

In this study, we predicted the continent-of-origin for oak trees with high accuracy. “Continent-of-origin” is a broad definition for geographic origin, but it is relevant to laws pertaining to commercial trade of white oak wood. The white oaks include some of the most important hardwoods for flooring and furniture, and represents the species with the highest export volume from the U.S. [24]. The generic trade name “white oak” applies to over a dozen of the 50+ known species from *Quercus* sect. *Quercus*, and they are geographically distributed across North America, Europe and Asia [45]. One species from this group – *Q. mongolica* – is protected by the Convention on International Trade in Endangered Species Appendix III as regionally threatened due to pressure from illegal logging (http://checklist.cites.org). Species of white oak cannot be discriminated using anatomy or chemistry, and this has allowed illegally-harvested *Q. mongolica* wood to be mixed with legally-sourced white oak wood in the commercial product stream [25]. For white oaks, quickly and accurately determining the geographic source of wood to country, continent, or hemisphere would make it possible to independently validate claims of geographic origin and taxonomy for diverse wood products.

White oaks are notorious for exhibiting high intraspecific variation, low reproductive barriers, and genetic variation that transcends species boundaries [46, 47]. This makes white oaks an exceptionally challenging group to classify based on DNA variation. Evolutionary studies based on chloroplast and nuclear genome partitions have shown that the combined influences of hybridization, geographic isolation, and evolutionary divergence [28, 48, 49] have created a network of genealogies that cannot be translated into simple classifications [15, 50](e.g., DNA barcodes). The scale of ‘continent’ is where phylogenetic and geographic signals show the greatest congruence in white oaks [28], and this is the signal we are able to capture with our alignment-free method. In this particular case, the taxonomic identity of samples within the group of white oaks cannot be determined, but the continental geographic origin can be determined with great accuracy. In less complex biota than the genus *Quercus,* the ${d}_2^{\ast }$–based KNN prediction approach may be informative at finer geographic scales (e.g., specific countries, provinces, or conservation reserves), and it has the potential to be extended to determining taxonomic identity.

The fact that our approach works so well for differentiating white oaks, one of the most complicated groups of trees, is strong indication that it has wider utility for determining the geographic origin and taxonomic identity of a broader array of biological samples. Not only is our approach effective, it can also be implemented uniformly across any taxon that can be sampled by shotgun sequencing or ‘genome skimming’ [51]. The practical implications of this are enormous, given the recent rapid growth in DNA sequencing capacity, as well as the massive scale of commerce involving biological material and the high prevalence of provenance and taxonomic mislabeling. The improvements in identification described here can directly aid ongoing domestic and international efforts to improve legality, an important facet of sustainability.

## Methods

### NGS data from white oak samples

NGS whole genome shotgun (WGS) sequencing data of 99 white oaks from North America, Europe and Asia were downloaded from NCBI BioProject PRJNA269970. The sequence data for these samples was derived from leaf tissue sampled from specimens collected in the field, which were previously published [15]. Four samples (SRR2053123, SRR2053080, SRR2053066, SRR2053060) showed less than 8 Mbp sequence data and were discarded due to insufficient data. Two-dimensional PCoA of the 95 tree samples based on all six dissimilarity measures identified three samples (SRR2053124 [*Q. robur*], SRR2053125 [*Q. robur*], SRR2053082 [*Q. dentata*]) as extreme outliers (Additional File 5, Figure S1). These samples also had low sequence yields (237 Mbp, 274 Mbp, 473 Mbp), which could be indicative of poor library quality; for this reason, these samples were also removed from analysis, leaving 92 samples with sequence yields in the range of 360 Mbp to 1765 Mbp.

Mean dissimilarity measures used in this study are weakly and inversely correlated with sequence quantity. To reduce confounding effects caused by different sequence quantities, we down-sampled data for all 92 samples to produce three different datasets. Two datasets consisted of random samples of reads totaling to 50 Mbp and 100 Mbp for each sample, respectively. The third consisted of reads totaling to 300 Mbp. All samples were divided into three geographic categories based on their continental origin. Samples from the United States and Canada were categorized as North America (NA), samples from west of 60°E longitude were categorized as Europe (EU), and samples from east of 60°E longitude were categorized as Asia (AS).

### Dissimilarity measures between genomes based on NGS data

We used six alignment-free distance/dissimilarity measures based on the relative frequencies of *k*-mers (*k*-grams, *k*-tuples, *k*-words) to compare any pair of samples. These are the traditional Manhattan, Euclid, and *d*_2_ [26] distances, and three recently developed background-adjusted dissimilarity measures: CVTree [16], ${d}_2^{\ast }$ and ${d}_2^s$ [17–19]. Detailed definitions of these measures are given in Additional File 6. The background-adjusted dissimilarity measures are obtained based on a model of the background DNA sequence using an *m*-th order Markov chain, with *m* estimated using the method developed for NGS short read data [18]. For the available white oak NGS data, *m* = 10. Previous studies showed that ${d}_2^{\ast }$ and ${d}_2^s$ performed well when *k* = *m* + 2 [21]. Therefore, we used *k* = 12 and Markov order *m* = 10 to calculate the dissimilarity between pairs of samples. For comparison, we also used *k* = 12 in the calculation of the traditional Manhattan, Euclid and *d*_2_ distances. All calculations of the pairwise dissimilarity values were carried out using the software package CAFE [22], a user-friendly and efficient package for calculating 28 alignment-free sequence dissimilarity measures.

### Circular plots and principal coordinate analysis

For each sample, we found the most similar samples to it and linked them using the circular visualization tool [27] based upon each of the six pairwise distance/dissimilarity measures. Of the six dissimilarity measures, the circular plots of the 92 samples show that the most similar samples are from the same continent-of-origin using the ${d}_2^{\ast }$ and ${d}_2^s$dissimilarity, while others contain some mistakes. Since ${d}_2^{\ast }$ is simpler to calculate than ${d}_2^s$ and ${d}_2^s$ is more sensitive to sequencing platforms (Additional File 4, Figure S5(A)), we focused on ${d}_2^{\ast }$ for the remaining studies. Pairwise ${d}_2^{\ast }$ dissimilarities among the 92 white oak samples (33 samples from Asia, 16 samples from Europe, 43 samples from North America) of 50, 100, and 300 Mbp were used for principal coordinate analysis using R.

### Intra- and inter-continental ${\boldsymbol{d}}_{\mathbf{2}}^{\ast }$ dissimilarity distributions

For each quantity of sequence (50, 100 and 300 Mbp), we contrasted pairwise intra-continental dissimilarities with pairwise inter-continental dissimilarities. The hypothesis that intra-continental dissimilarities should be lower than inter-continental dissimilarities was tested with the Wilcoxon-Mann-Whitney (WMW) test statistic. To obtain a *p*-value, we permuted the continental labels of the tree samples 10^7^ times and then compared the intra- with inter-continental${d}_2^{\ast }$ dissimilarities using the WMW statistic for the permuted samples. We approximated the p-value by the fraction of times that the WMW values for the permuted data were higher than that for the original labelled data.

### Continental origin prediction by KNN and ${\boldsymbol{d}}_{\mathbf{2}}^{\ast }$

A K-nearest neighbors (KNN) algorithm was used to predict the continental origins of white oaks. For each quantity of sequence (50, 100 and 300 Mbp), samples were randomly divided into training and test data sets, with the training set making up 91, 77, 60, 45, 30 or 15 of the total 92 tree samples. For each sample in the test set, we found its K-nearest neighbors measured by ${d}_2^{\ast }$ in the training set and predicted its continental origin by a majority vote. One hundred distinct splits of the data into training and test data sets were constructed, for each of which the origin was predicted for a range of K from 1 to 10.

To investigate the effects of sequencing error on the prediction accuracy of KNN, we randomly mutated the sequences by altering individual nucleotides at a rate of 5%; erroneous bases were selected with equal probability without regard to transition/transversion bias or regional nucleotide composition. We recalculated ${d}_2^{\ast }$ dissimilarities between test samples and training samples and then calculated the prediction accuracy, and repeated the process of evaluating the KNN prediction accuracy 100 times. We then compared the average KNN prediction accuracy with simulated errors to the KNN prediction accuracy without simulated errors; both cases included the non-zero background of naturally occurring errors.

### Effect of reference tree on geographic prediction accuracy

For each DNA sample, the predicted continental origin depends on the identity of reference trees and the NGS data obtained from the query sample. We quantified variation in prediction due to each of these sources using random resampling. First, we created 1000 sets of reference tree samples, each consisting of a random selection with replacement of 92 reference tree samples from the original 92 samples. Continental origin was predicted with K = 1 and K = 3. The reference-confidence (RC) was defined as the fraction of times that the predicted continental origin is consistent with that using all 92 original reference tree samples.

To quantify the variation resulting from NGS data derived from the unknown samples, we created 10 samples from the data by sampling reads to a total of 100 Mbp per sample without replacement. The NGS-confidence (NC) is defined as the fraction of times the predicted continental origin is the same as that for the most commonly predicted origin among the 10 runs.

### Effect of laboratory and sequencer error on accuracy

To test if our computational method of predicting continental origin is sensitive to variation in NGS library construction method or sequencing platform, we assembled data from other genomics studies of white oaks that were unrelated to the training data set, and used the data to predict continent-of-origin for each sample. For each of these comparisons, we randomly chose 100 Mbp from each data set, calculated the ${d}_2^{\ast }$ dissimilarity between these datasets and the 92 samples in our reference data set, and used KNN to predict the continental origins of the test samples. Three data sets were used and the characteristics of all samples are given in Additional file 7 (Table S1).

(A) Total genomic data derived from one North American California Valley Oak (*Q. lobata* Nee), a white oak member from Sect. *Quercus* (https://www.ncbi.nlm.nih.gov/bioproject/308314; https://valleyoak.ucla.edu) [52]. Samples were sequenced using Illumina HiSeq2500 with different library preparation methods and read lengths than those used to construct our reference library. These data allowed us to test whether different library construction methods produce accurate geographic predictions.
(B) Total genomic data derived from one European Swiss Pedunculate Oak (*Q. robur* L.), another white oak member from Sect. *Quercus* (https://www.ncbi.nlm.nih.gov/bioproject/327502). DNA was isolated from leaves of two branches of a 234-year-old oak tree [53]. This project includes 30 SRA experiments, 8 using the Illumina HiSeq 2000 (similar to our reference data), and 22 using long single-molecule (2489 bp to 7622 bp) real-time sequencing from the PacBio-SMRT platform. These data allow us to test whether different sequencing platforms with different error profiles produce accurate geographic predictions.
(C) Targeted genomic data derived from a restriction site-associated DNA Sequencing (*RAD*-*Seq*) [31] study of multiple white oaks [32]. This study used the restriction enzyme *PstI* to selectively enrich genomic regions for targeted Illumina sequencing. For our study, we predicted the continental origins of seven white oak samples based on *RAD-Seq* data: North America (*Q. bicolor*: SRR5632514, *Q. stellata*: SRR5632513, *Q. lobata*: SRR5632586), Europe (*Q. robur*: SRR5632600, *Q. petraea*: SRR5284338), and Asia (*Q. dentata*: SRR5632587, *Q. mongolica*: SRR5284345). Importantly, two of these *RAD-Seq* samples were derived from the identical DNA preparation from single trees that were used in our reference database of shotgun DNA sequences (*Q. petraea*: shotgun library SRR2053073; *Q. mongolica*: shotgun library SRR2053072) [15, 28].

## Additional files

Additional file 1:**Figure S2.** The circular plots of 92 white oak tree samples based on the six dissimilarity measures: ${d}_2^{\ast }$, ${d}_2^S$, CVTree, Euclidean, and Manhattan, using (A) 50 Mbp and (B) 300 Mbp of next generation sequencing data. Different sectors correspond to different continents, with NA in red, EU in orange and AS in blue. Within each sector, samples are sorted by their longitude, so that samples that are geographically close are also close to each other in the figure. The most similar tree samples to each sample are linked. The k-mer length is 12 and the Markov order of the background sequence is 10 for ${d}_2^{\ast }$, ${d}_2^S$ and CVTree. The most similar samples to each sample according to ${d}_2^{\ast }$ and ${d}_2^S$are from the same continent-of-origin. (PDF 671 kb)

Additional file 2:**Figure S3.** The relationship between the first three principal coordinates and (A) longitude and (B) latitude of the tree samples based on the ${d}_2^{\ast }$ dissimilarity values using sequencing quantity of 100 Mbp. The *k*-mer length is 12 and the Markov order of the background sequence is 10. The first principal coordinate separates the North America tree samples from the Europe and Asia tree samples, and the third principal coordinate separates the Europe samples from Asia samples. The second principal coordinates of most Asian samples are larger than that of the Europe samples. However, the second principal coordinate does not separate them. (PDF 68 kb)

Additional file 3:**Figure S4.** Comparison of intra- and inter-continental ${d}_2^{\ast }$ dissimilarities with sequence quantity of 50 Mbp and 300 Mbp, based on comparisons to a) Asian, b) European, and c) North American sources. The *k*-mer length is 12 and the Markov order of the background sequence is 10. The *p*-values were calculated based on the Wilkinson-Mann-Whitney test statistic and by permuting the continental labels of the white oak tree samples 10^7^ times. The inter-continental ${d}_2^{\ast }$ dissimilarities are significantly higher than intra-continental ${d}_2^{\ast }$ dissimilarities. (PDF 170 kb)

Additional file 4:**Figure S5.** The circular plots for independent samples sequenced using a) Illumina NGS of a California Valley Oak tree, b) a mixture of short- and long read from with both Illumina and PacBio sequencing of the Pendunculate Oak tree, and c) seven diverse tree samples using RAD-seq. The (A) ${d}_2^S$ dissimilarity and (B) Manhattan distance measures of each independent sample with the 92 reference samples were calculated and the two most similar reference samples are linked. (PDF 225 kb)

Additional file 5:**Figure S1.** The two-dimensional principal coordinate (PCoA) plots of the 95 tree samples based on the Euclidean distance (Eu), Manhattan distance (Ma), d_2_ dissimilarity, CVTree, ${d}_2^S$ and ${d}_2^{\ast }$ of the samples for different sequence quantities of 50, 100 and 300 Mbp, respectively. Three outliers, SRR2053124 [*Q. robur*], SRR2053125 [*Q. robur*], SRR2053082 [*Q. dentata*], were identified. However, the other samples cluster together. (PDF 224 kb)

Additional file 6: Details on the definitions of six alignment-free distance/dissimilarity measures between two genomes based on NGS data. (PDF 123 kb)

Additional file 7: **Table S1.** Library construction, sequencing, and genome enrichment methods used for all DNA libraries in this study. (PDF 29 kb)

## Abbreviations

AS: Asia
EU: Europe
KNN: K nearest neighbors
NA: North America
NC: NGS-confidence
NGS: Next generation sequencing
RAD*-*Seq: restriction site-associated DNA sequencing
RC: reference confidence
WGS: whole genome shotgun sequencing
WMW: Wilcoxon-Mann-Whitney test
