# Supplementary material for: Alignment-free genome comparison enables accurate geographic sourcing of white oak DNA
Source: BMC Genomics. 2018 Dec 10;19:896. doi: 10.1186/s12864-018-5253-1 (PMC6288960; doi:10.1186/s12864-018-5253-1)
Supplement: Supplementary file 1 — Figure S2. The circular plots of 92 white oak tree samples based on the six dissimilarity measures: ${d}_2^{\ast }$d2∗, ${d}_2^S$d2S, CVTree, Euclidean, and Manhattan, using (A) 50 Mbp and (B) 300 Mbp of next generation sequencing data. Different sectors correspond to different continents, with NA in red, EU in orange and AS in blue. Within each sector, samples are sorted by their longitude, so that samples that are geographically close are also close to each other in the figure. The most similar tree samples to each sample are linked. The k-mer length is 12 and the Markov order of the background sequence is 10 for ${d}_2^{\ast }$d2∗, ${d}_2^S$d2S and CVTree. The most similar samples to each sample according to ${d}_2^{\ast }$d2∗ and [file 12864_2018_5253_MOESM1_ESM.pdf]

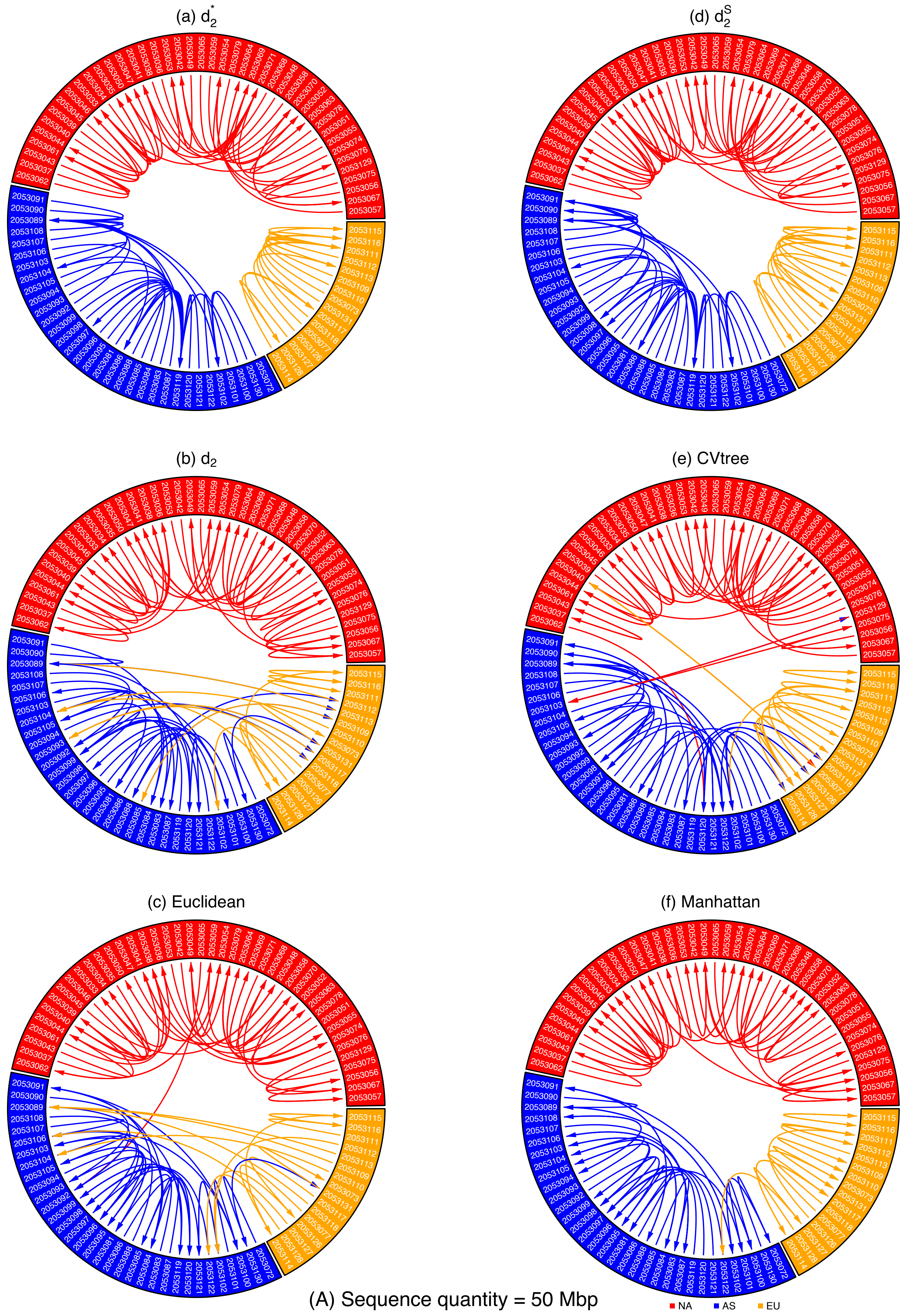

(A) Sequence quantity = 50 Mbp

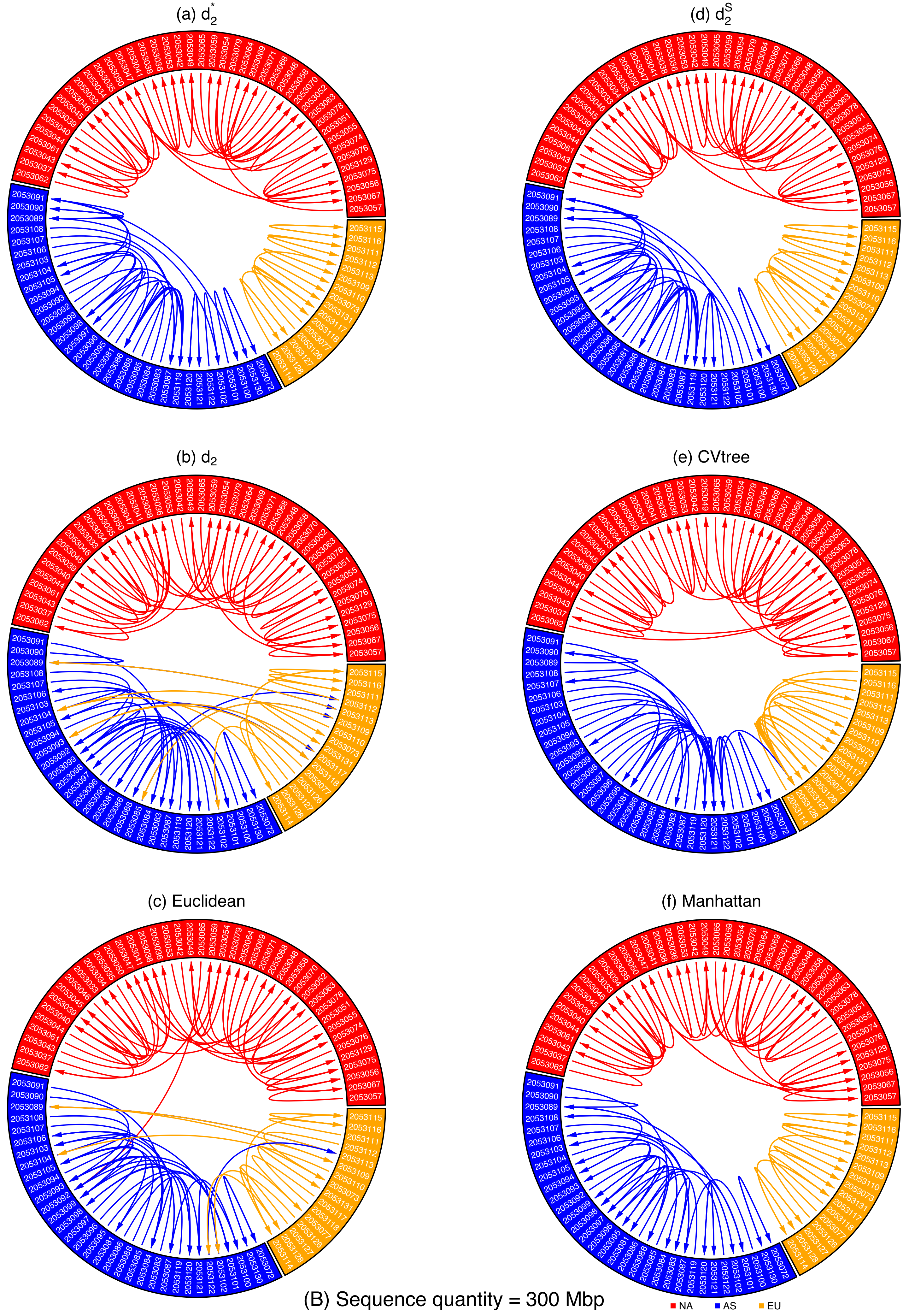

(B) Sequence quantity = 300 Mbp
