# Supplementary figures and images for: Alignment-free genome comparison enables accurate geographic sourcing of white oak DNA
Source: BMC Genomics. 2018 Dec 10;19:896. doi: 10.1186/s12864-018-5253-1 (PMC6288960; doi:10.1186/s12864-018-5253-1)

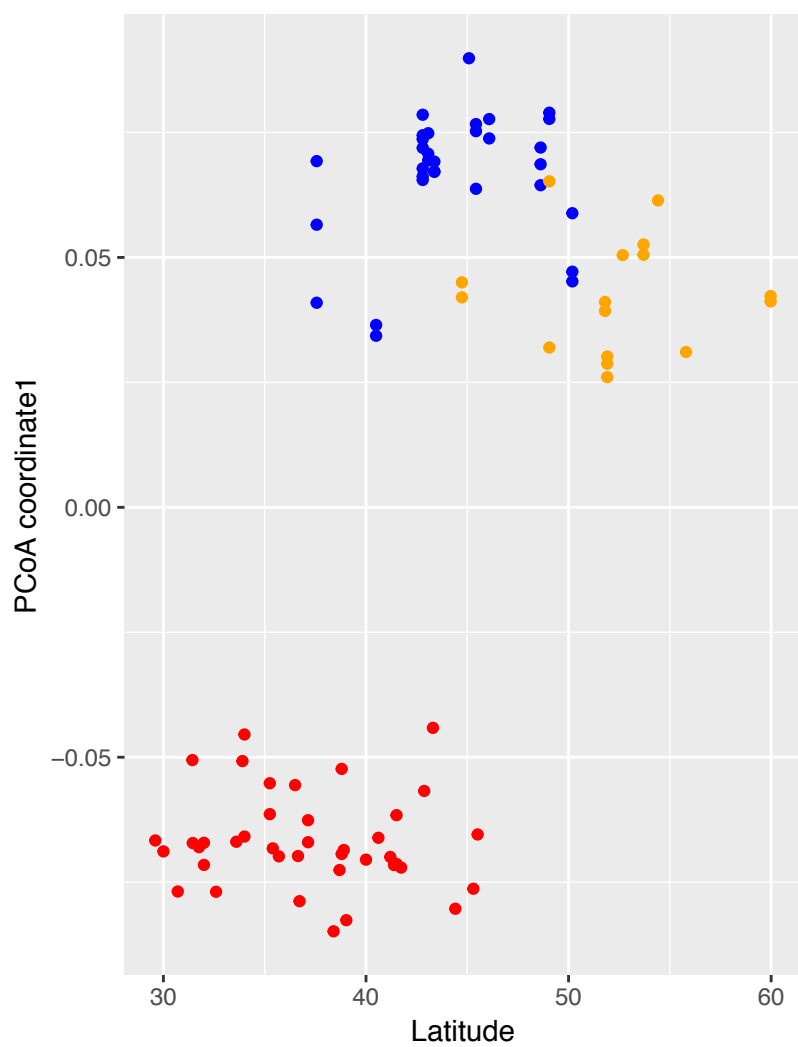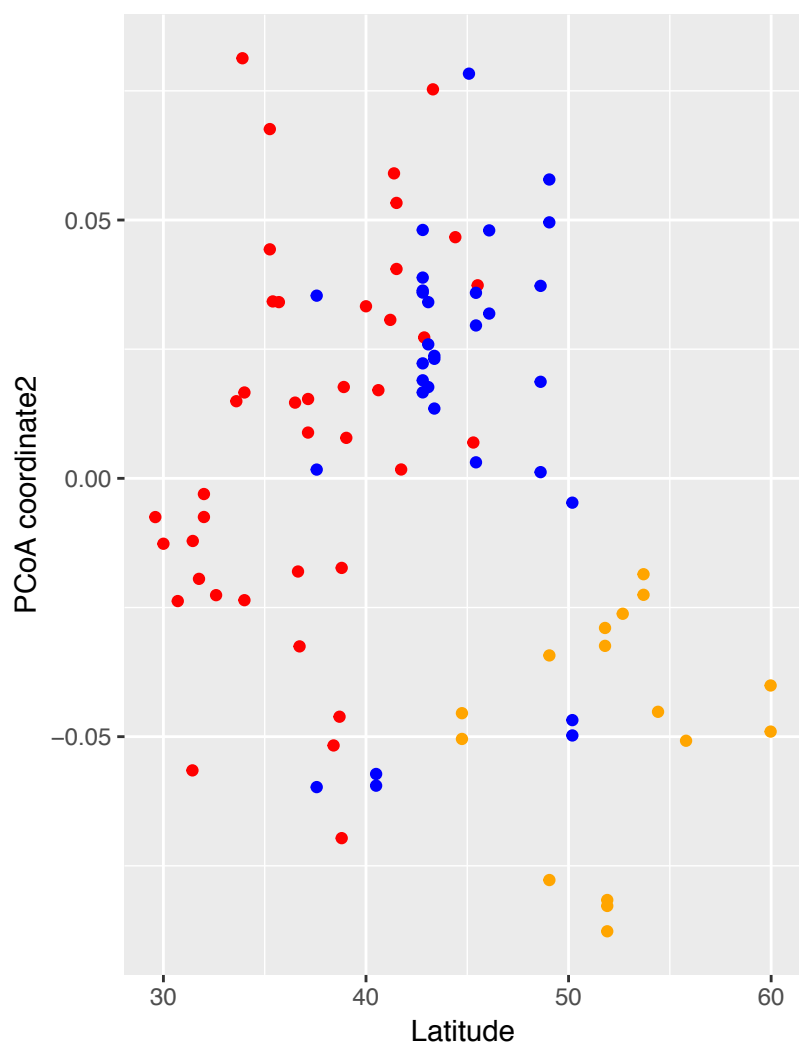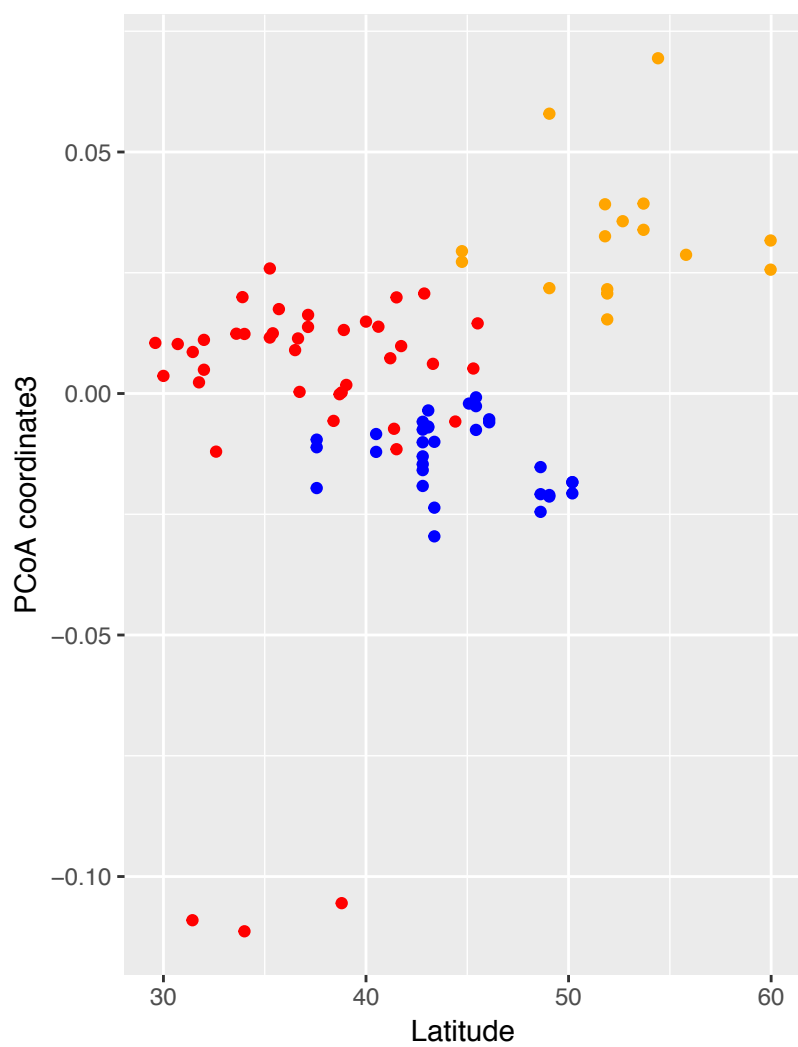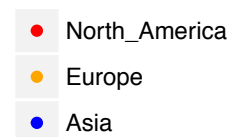

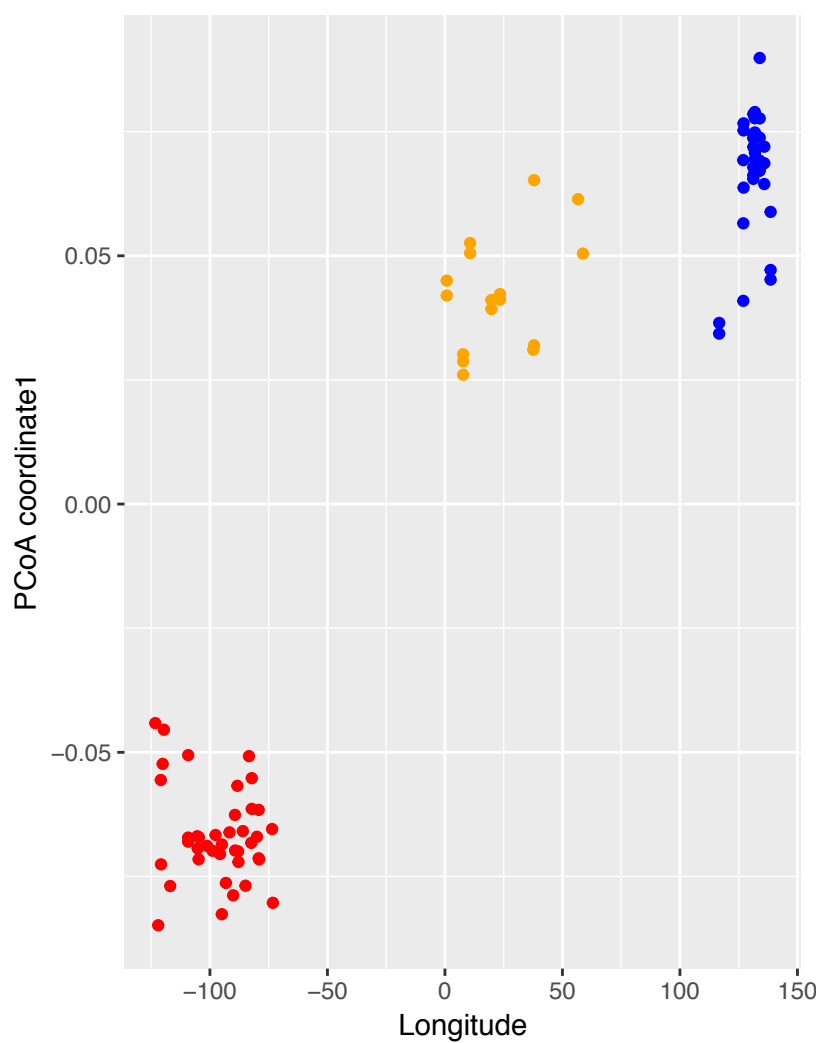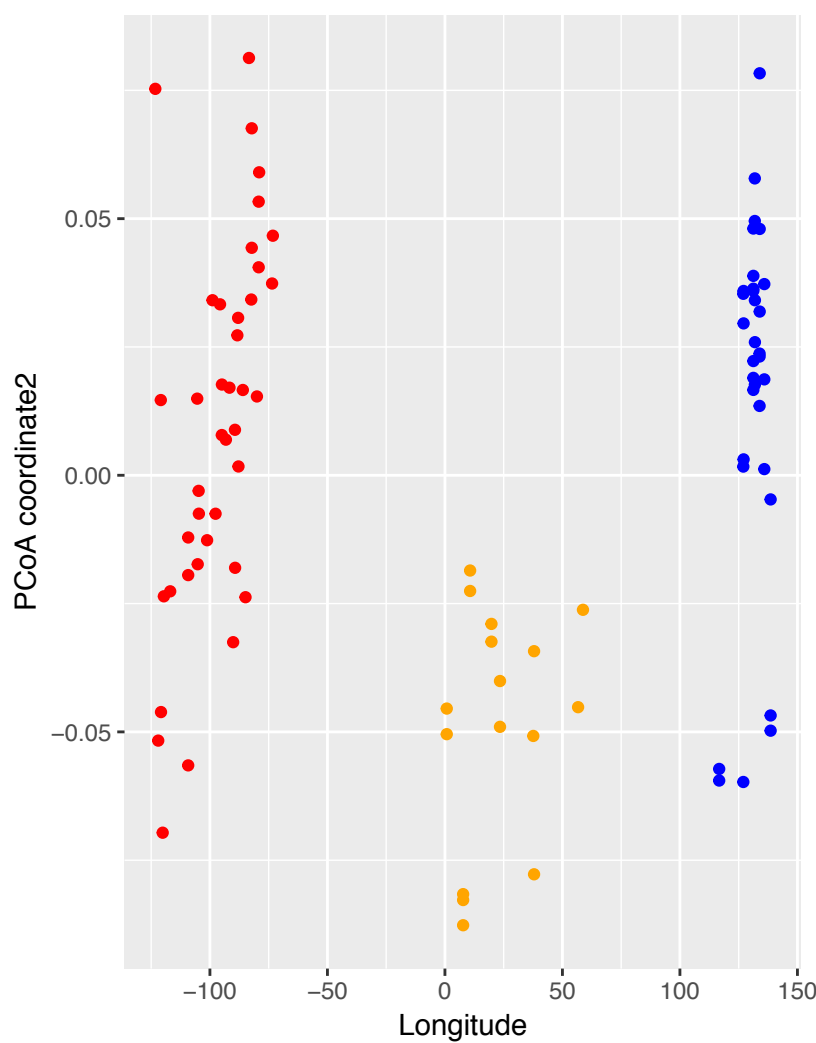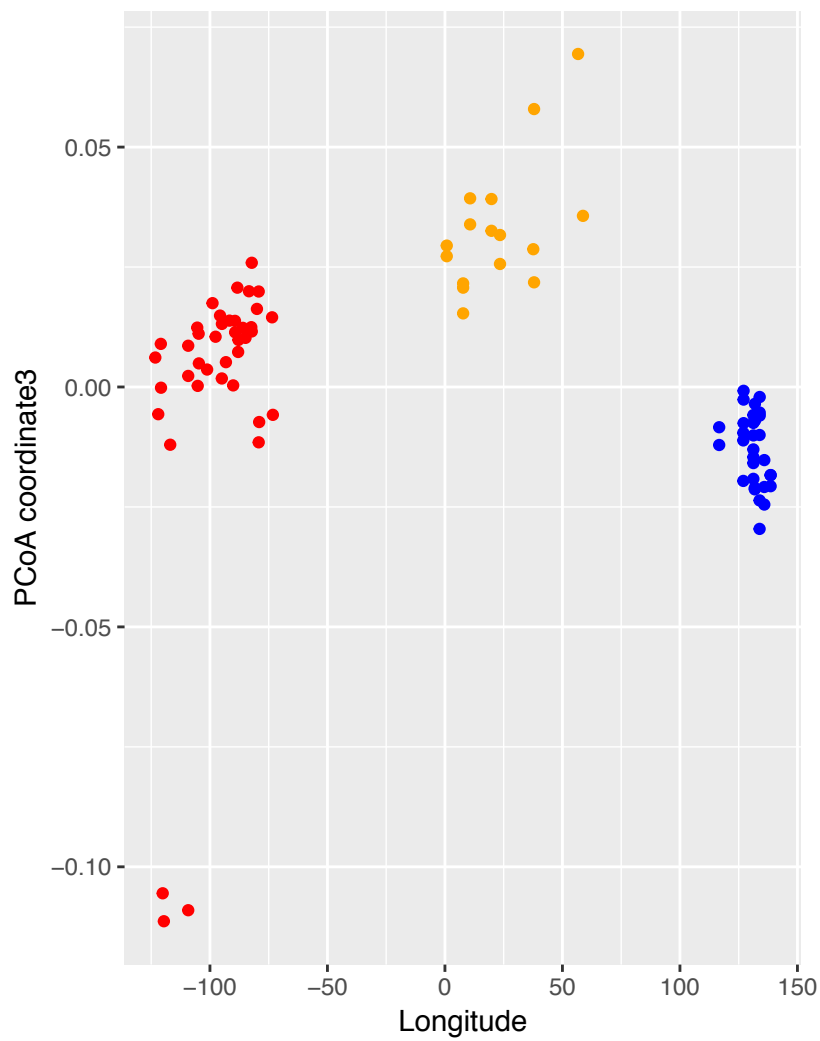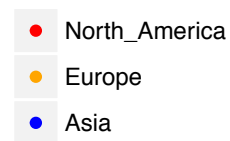

Supplement: Supplementary file 2 — Figure S3. The relationship between the first three principal coordinates and (A) longitude and (B) latitude of the tree samples based on the ${d}_2^{\ast }$d2∗ dissimilarity values using sequencing quantity of 100 Mbp. The k-mer length is 12 and the Markov order of the background sequence is 10. The first principal coordinate separates the North America tree samples from the Europe and Asia tree samples, and the third principal coordinate separates the Europe samples from Asia samples. The second principal coordinates of most Asian samples are larger than that of the Europe samples. However, the second principal coordinate does not separate them. (PDF 68 kb) [file 12864_2018_5253_MOESM2_ESM.pdf]

(A) Sequence quantity = 50 Mbp

(a)

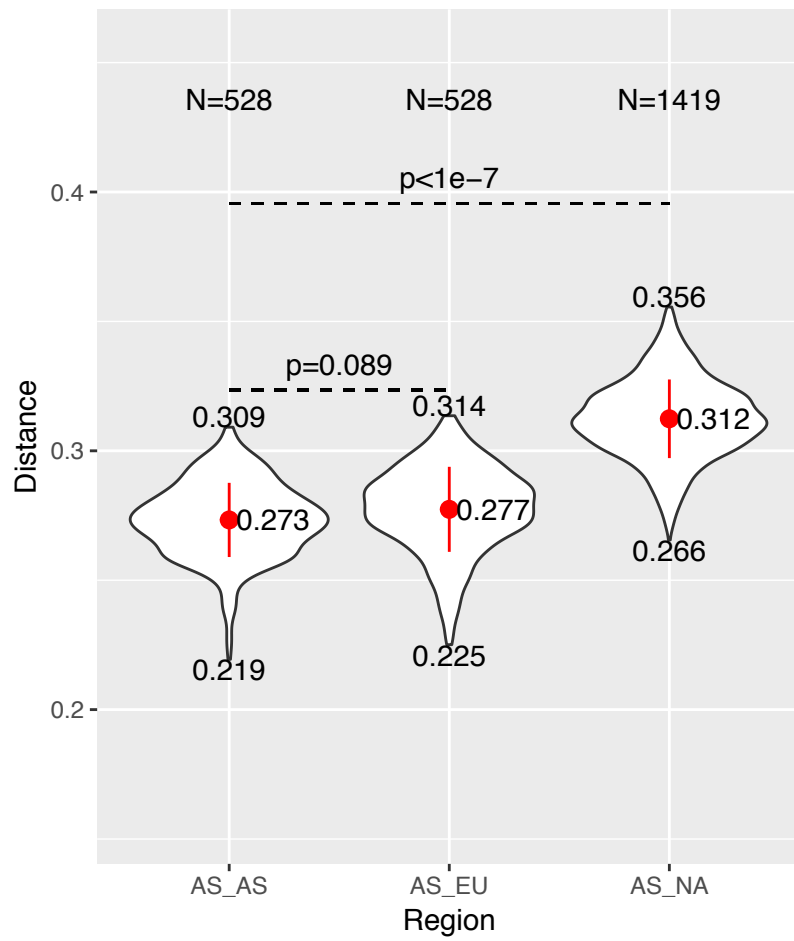

(b)

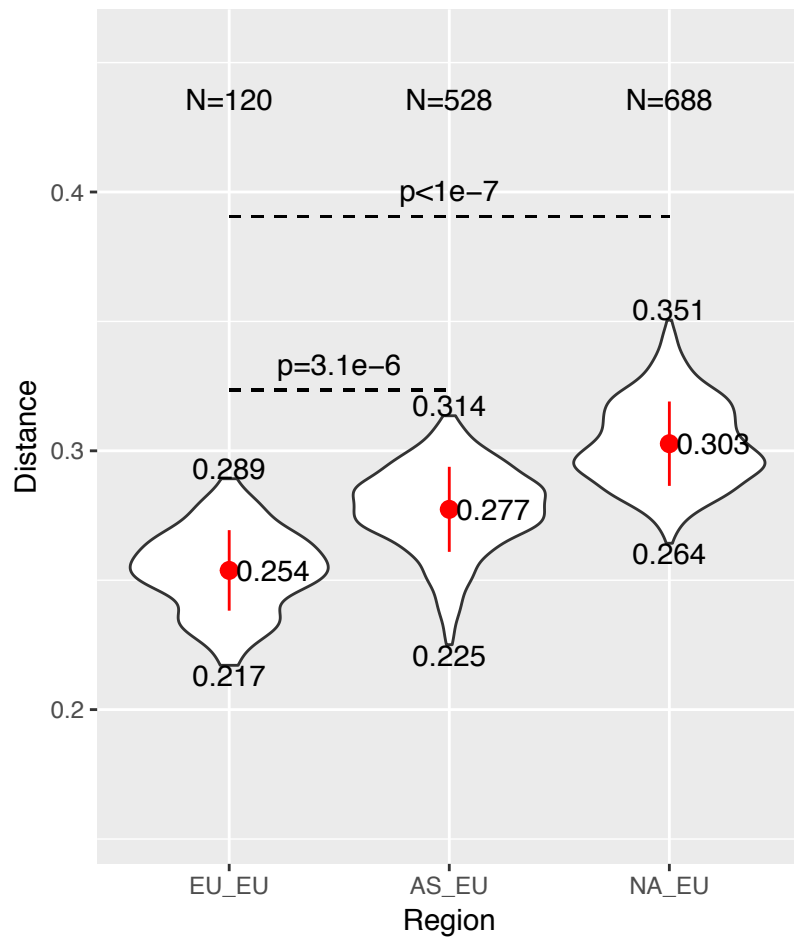

(c)

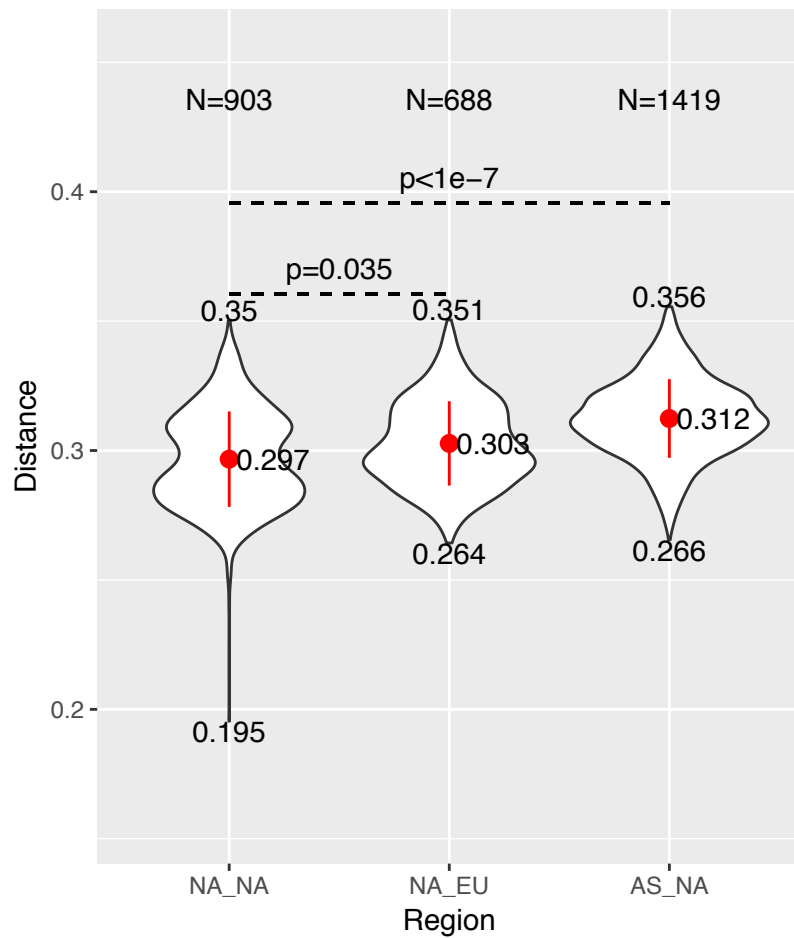

(B) Sequence quantity = 300 Mbp

(a)

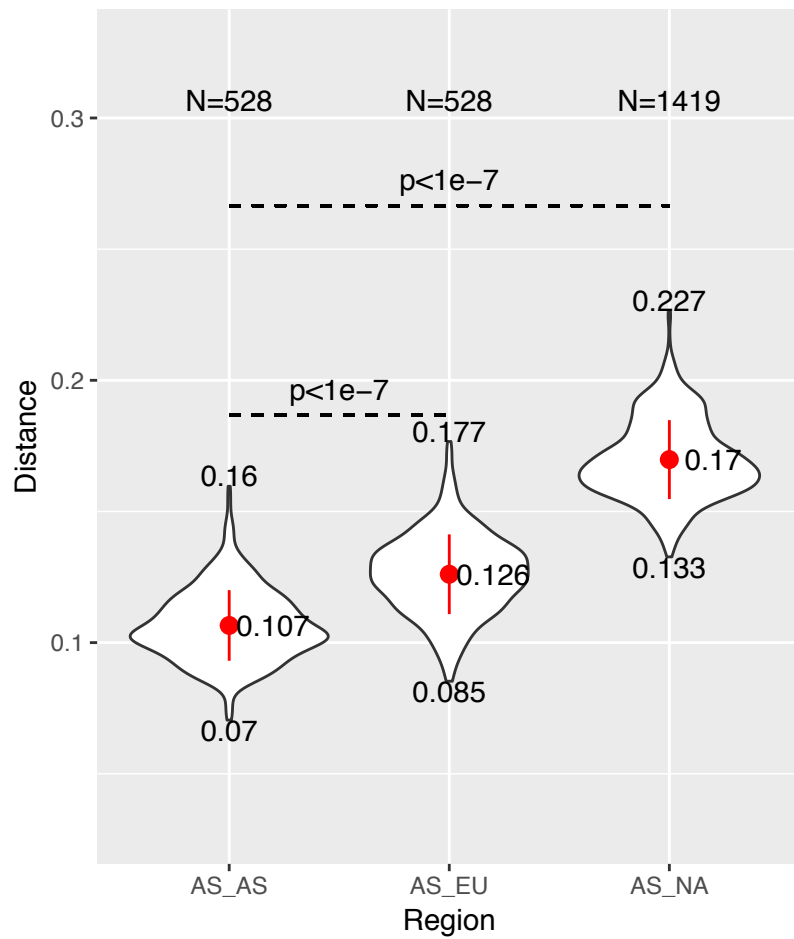

(b)

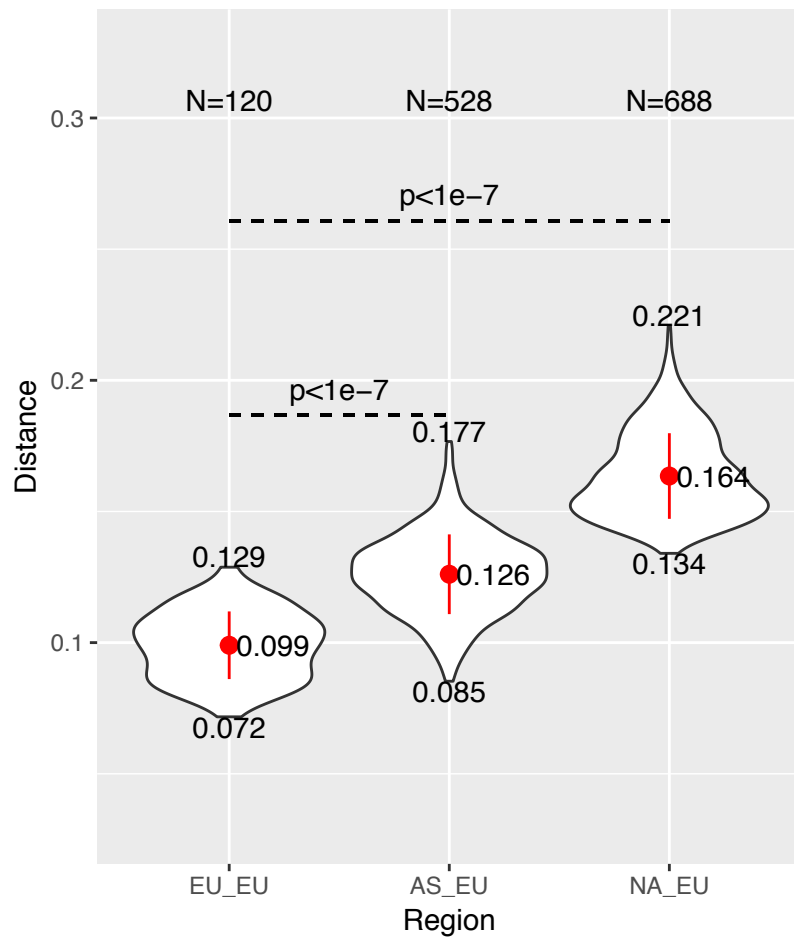

(c)

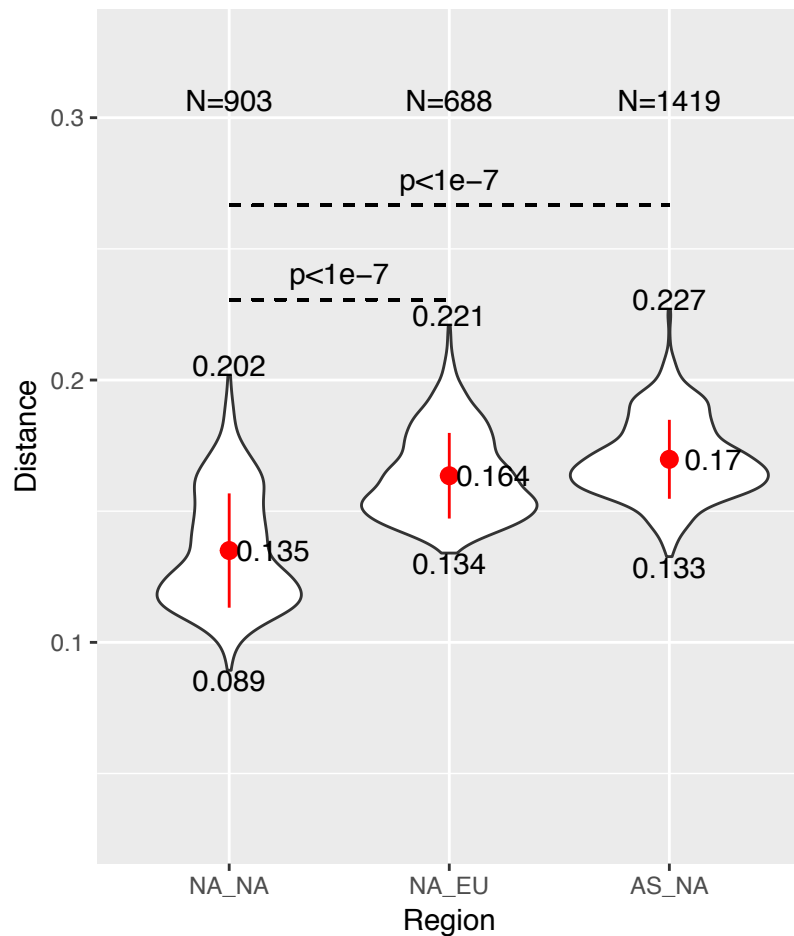

Supplement: Supplementary file 3 — Figure S4. Comparison of intra- and inter-continental ${d}_2^{\ast }$d2∗ dissimilarities with sequence quantity of 50 Mbp and 300 Mbp, based on comparisons to a) Asian, b) European, and c) North American sources. The k-mer length is 12 and the Markov order of the background sequence is 10. The p-values were calculated based on the Wilkinson-Mann-Whitney test statistic and by permuting the continental labels of the white oak tree samples 107 times. The inter-continental ${d}_2^{\ast }$d2∗ dissimilarities are significantly higher than intra-continental ${d}_2^{\ast }$d2∗ dissimilarities. (PDF 170 kb) [file 12864_2018_5253_MOESM3_ESM.pdf]
