# Supplementary material for: Alignment-free genome comparison enables accurate geographic sourcing of white oak DNA
Source: BMC Genomics. 2018 Dec 10;19:896. doi: 10.1186/s12864-018-5253-1 (PMC6288960; doi:10.1186/s12864-018-5253-1)
Supplement: Supplementary file 4 — Figure S5. The circular plots for independent samples sequenced using a) Illumina NGS of a California Valley Oak tree, b) a mixture of short- and long read from with both Illumina and PacBio sequencing of the Pendunculate Oak tree, and c) seven diverse tree samples using RAD-seq. The (A) ${d}_2^S$d2S dissimilarity and (B) Manhattan distance measures of each independent sample with the 92 reference samples were calculated and the two most similar reference samples are linked. (PDF 225 kb) [file 12864_2018_5253_MOESM4_ESM.pdf]

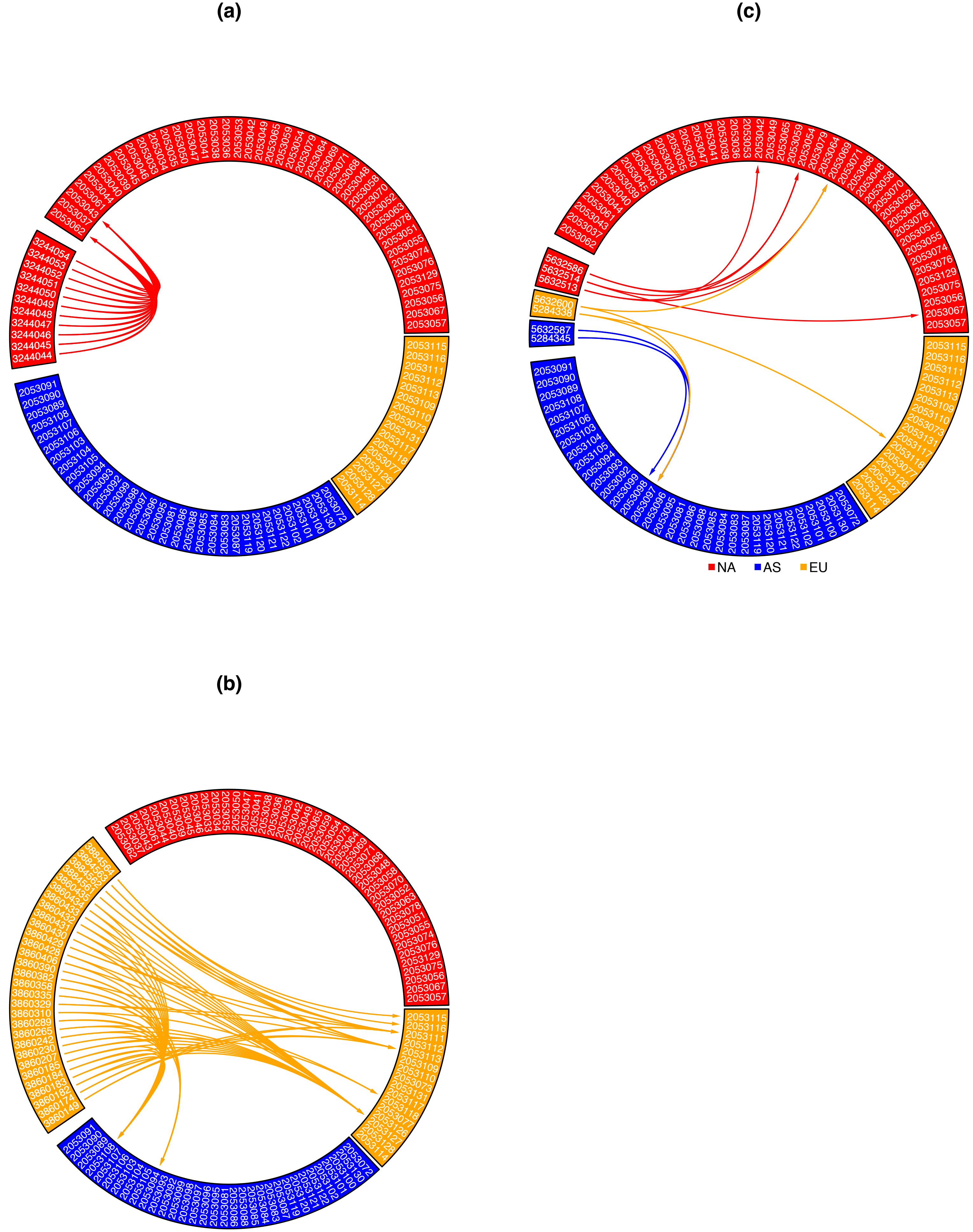

(A) Circular plots using the d_2^S dissimilarity

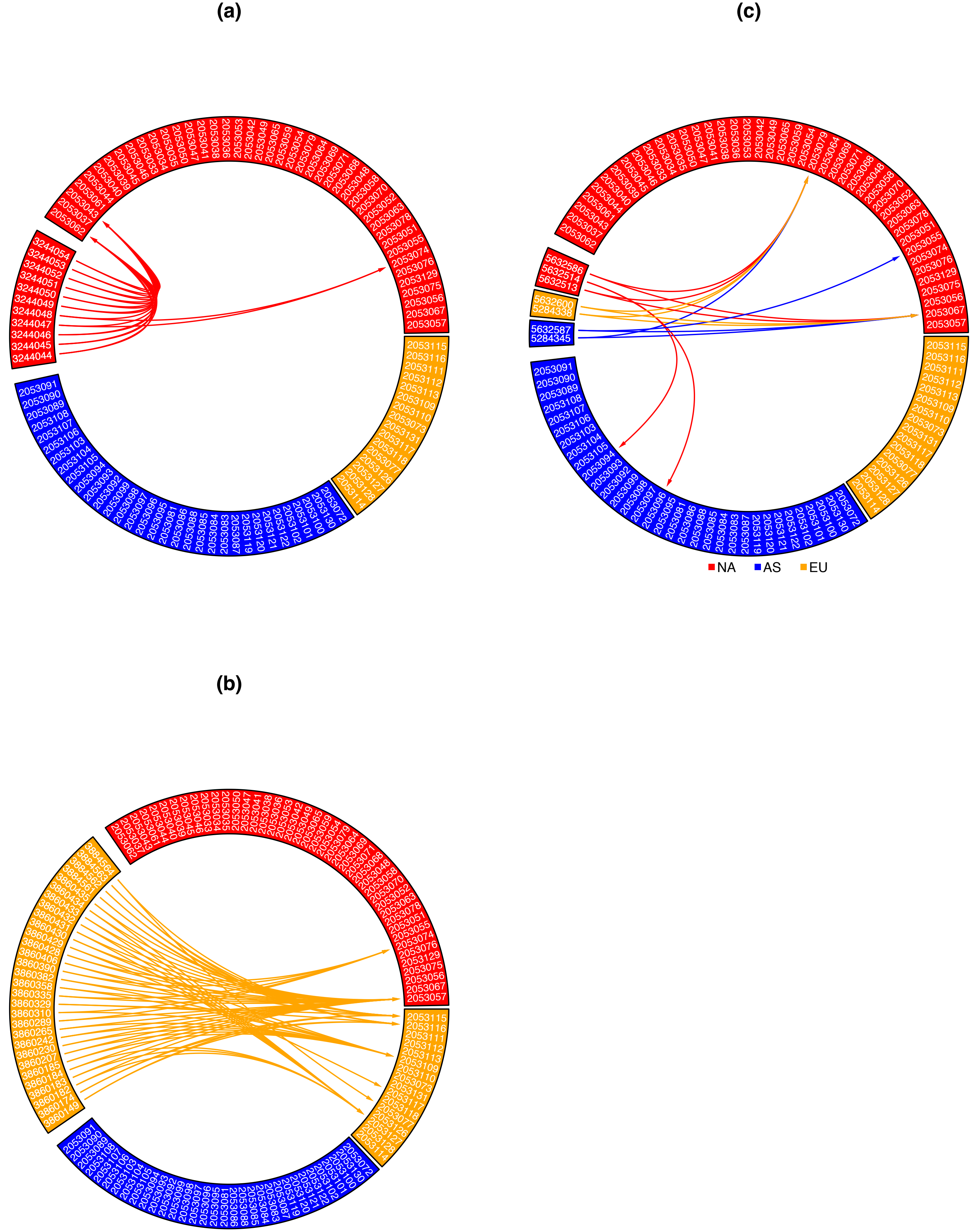

(B) Circular plots using the Manhattan distance
