# Supplementary material for: Alignment-free genome comparison enables accurate geographic sourcing of white oak DNA
Source: BMC Genomics. 2018 Dec 10;19:896. doi: 10.1186/s12864-018-5253-1 (PMC6288960; doi:10.1186/s12864-018-5253-1)
Supplement: Supplementary file 5 — Figure S1. The two-dimensional principal coordinate (PCoA) plots of the 95 tree samples based on the Euclidean distance (Eu), Manhattan distance (Ma), d2 dissimilarity, CVTree, ${d}_2^S$d2S and ${d}_2^{\ast }$d2∗ of the samples for different sequence quantities of 50, 100 and 300 Mbp, respectively. Three outliers, SRR2053124 [Q. robur], SRR2053125 [Q. robur], SRR2053082 [Q. dentata], were identified. However, the other samples cluster together. (PDF 224 kb) [file 12864_2018_5253_MOESM5_ESM.pdf]

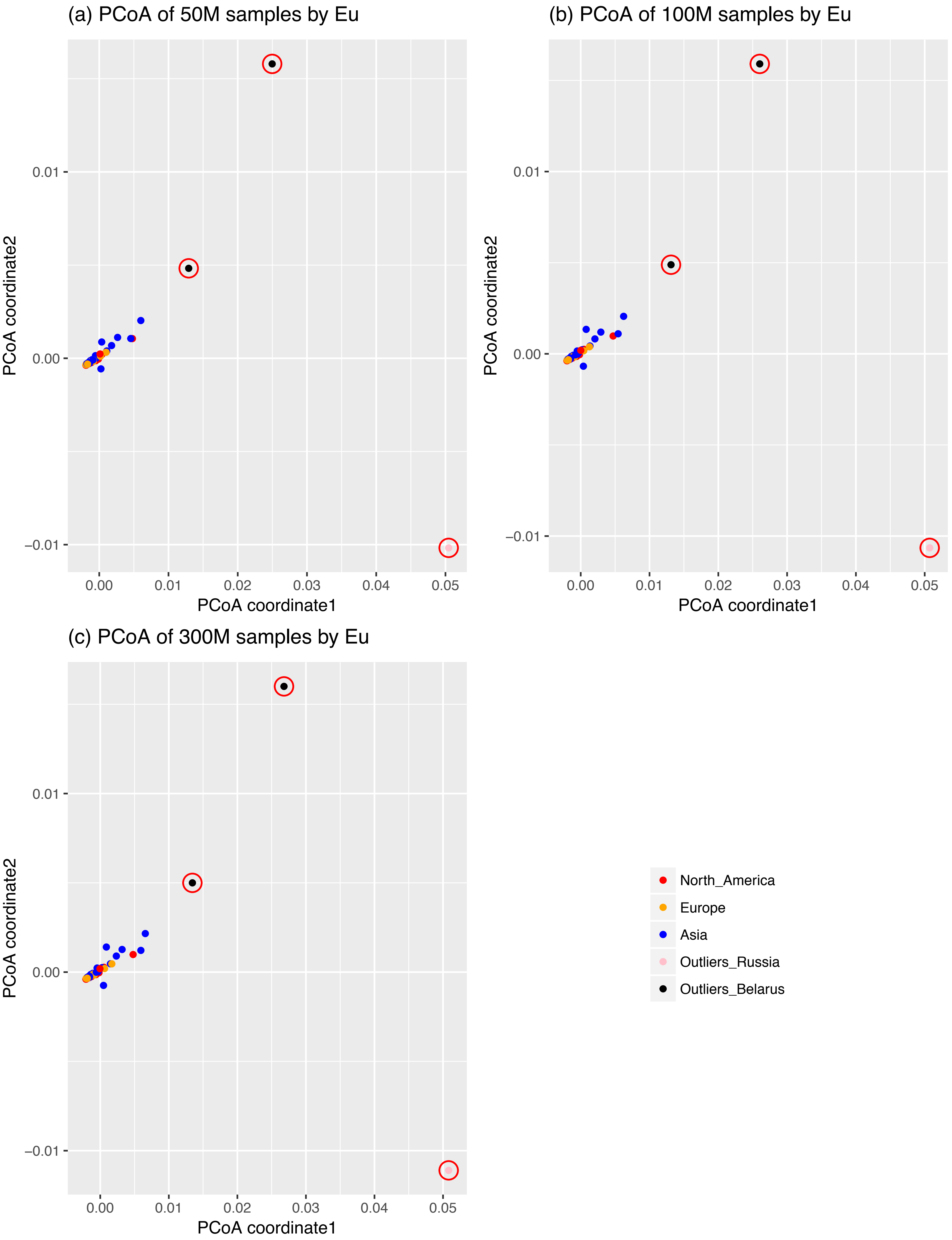

(a) PCoA of 50M samples by Eu
(b) PCoA of 100M samples by Eu
(c) PCoA of 300M samples by Eu
PCoA coordinate2
PCoA coordinate1
0.01
0.00
−0.01
0.00
0.01
0.02
0.03
0.04
0.05
North_America
Europe
Asia
Outliers_Russia
Outliers_Belarus

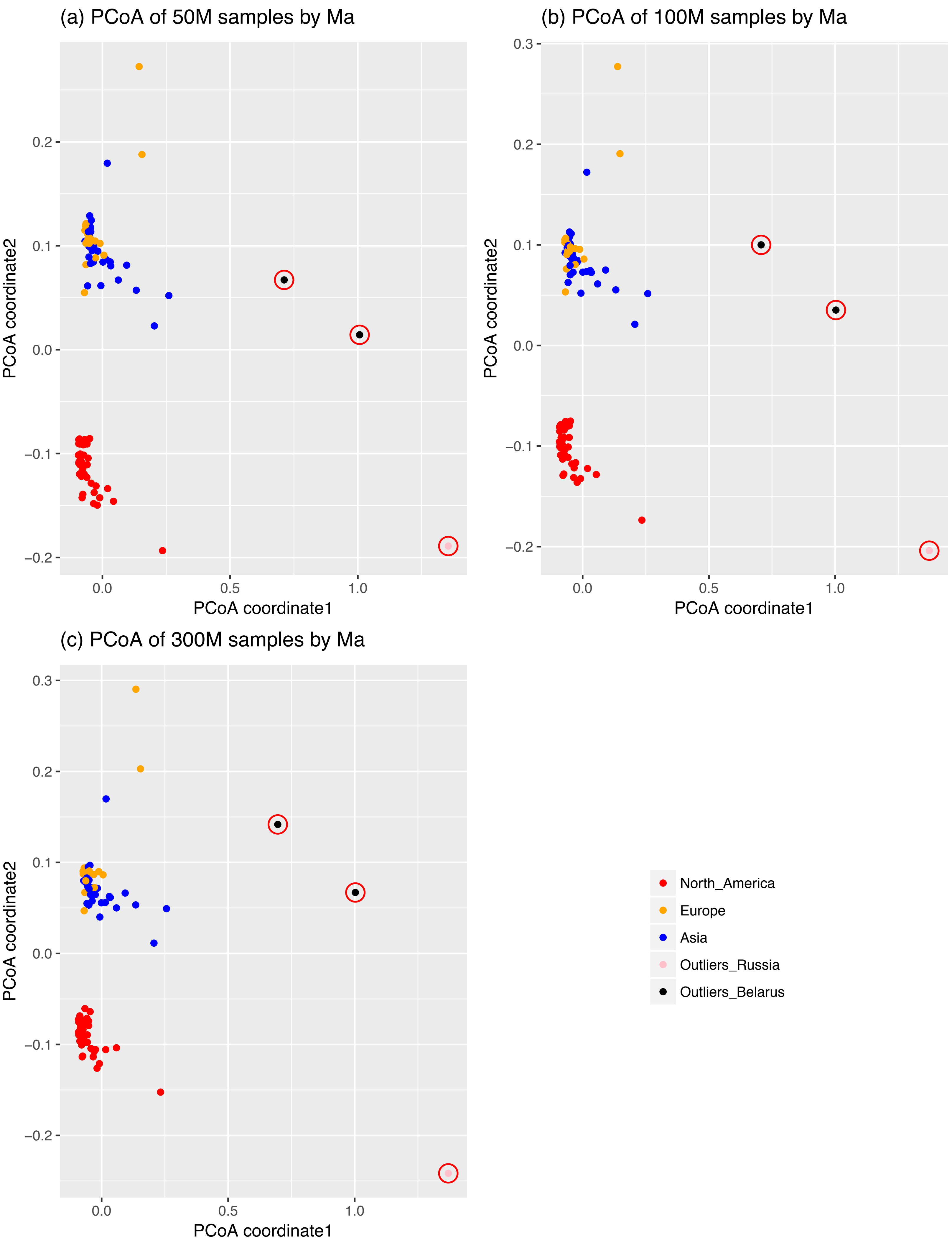
(a) PCoA of 50M samples by Ma
PCoA coordinate2
PCoA coordinate1
0.2
0.1
0.0
−0.1
−0.2
0.0
0.5
1.0
(b) PCoA of 100M samples by Ma
PCoA coordinate2
PCoA coordinate1
0.3
0.2
0.1
0.0
−0.1
−0.2
0.0
0.5
1.0
(c) PCoA of 300M samples by Ma
PCoA coordinate2
PCoA coordinate1
0.3
0.2
0.1
0.0
−0.1
−0.2
0.0
0.5
1.0
North_America
Europe
Asia
Outliers_Russia
Outliers_Belarus

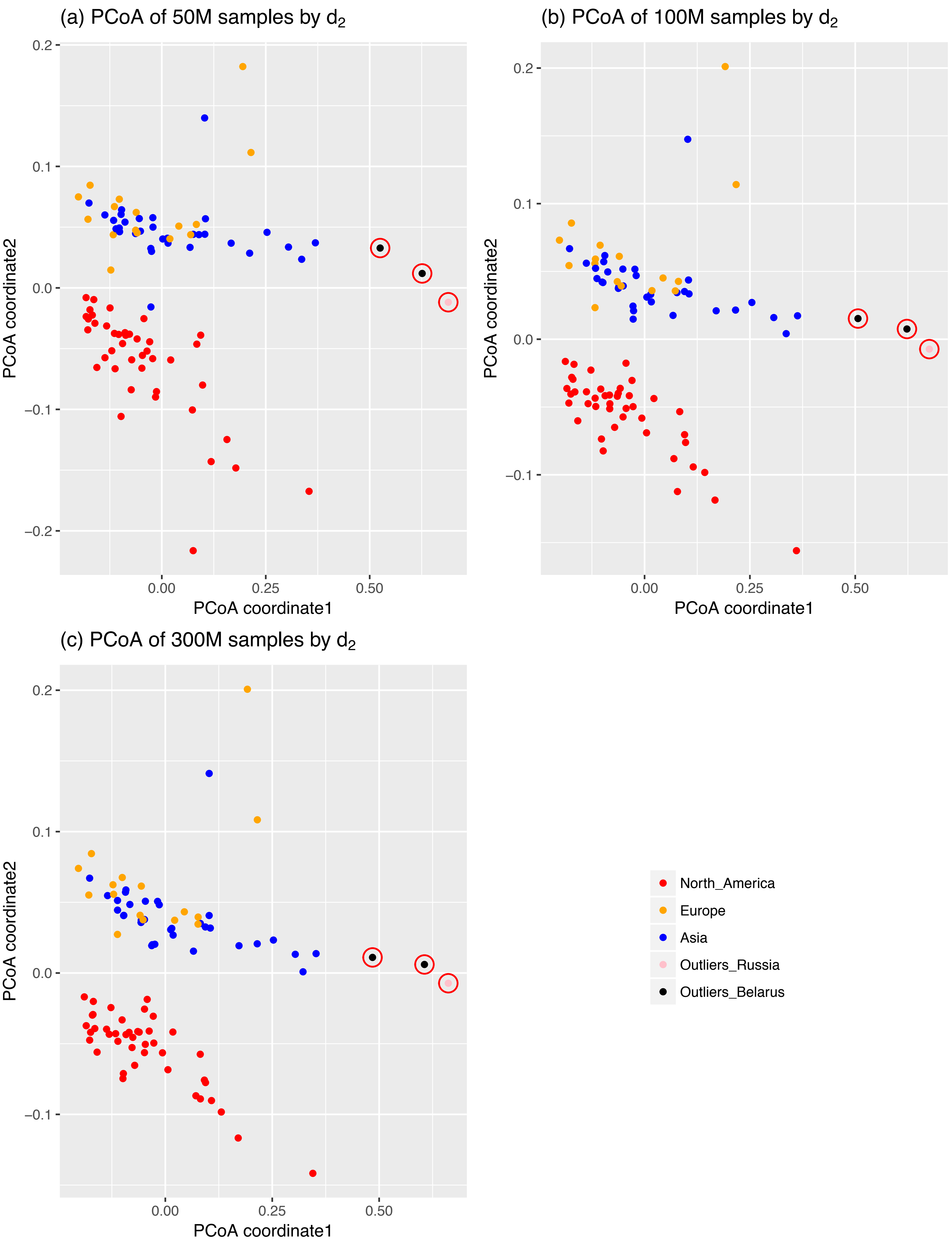
(a) PCoA of 50M samples by d_2
(b) PCoA of 100M samples by d_2
(c) PCoA of 300M samples by d_2
PCoA coordinate1
PCoA coordinate2
0.2
0.1
0.0
−0.1
−0.2
0.00
0.25
0.50
North_America
Europe
Asia
Outliers_Russia
Outliers_Belarus

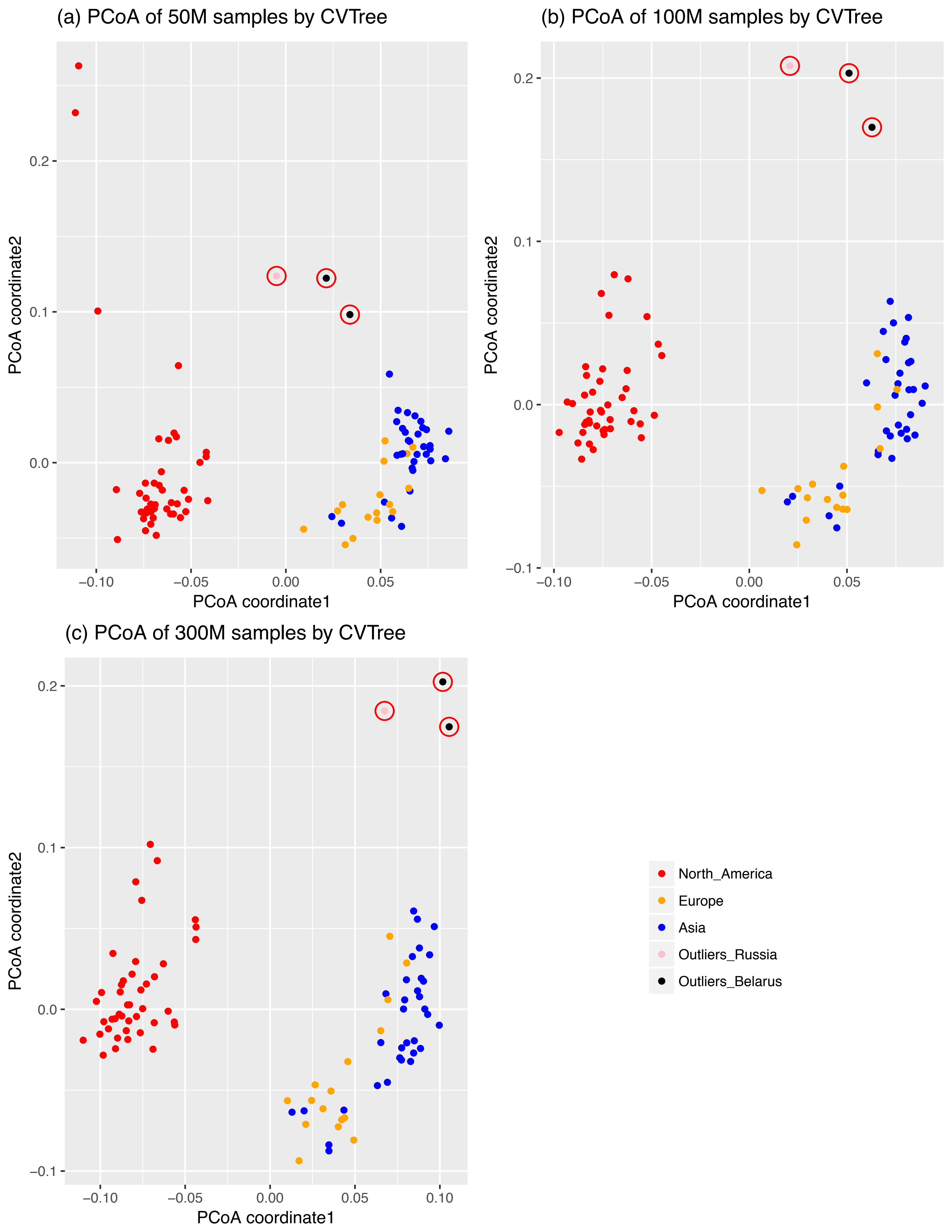

(a) PCoA of 50M samples by CVTree
(b) PCoA of 100M samples by CVTree
(c) PCoA of 300M samples by CVTree
PCoA coordinate1
PCoA coordinate2
0.2
0.1
0.0
−0.1
−0.10
−0.05
0.00
0.05
0.10
North_America
Europe
Asia
Outliers_Russia
Outliers_Belarus

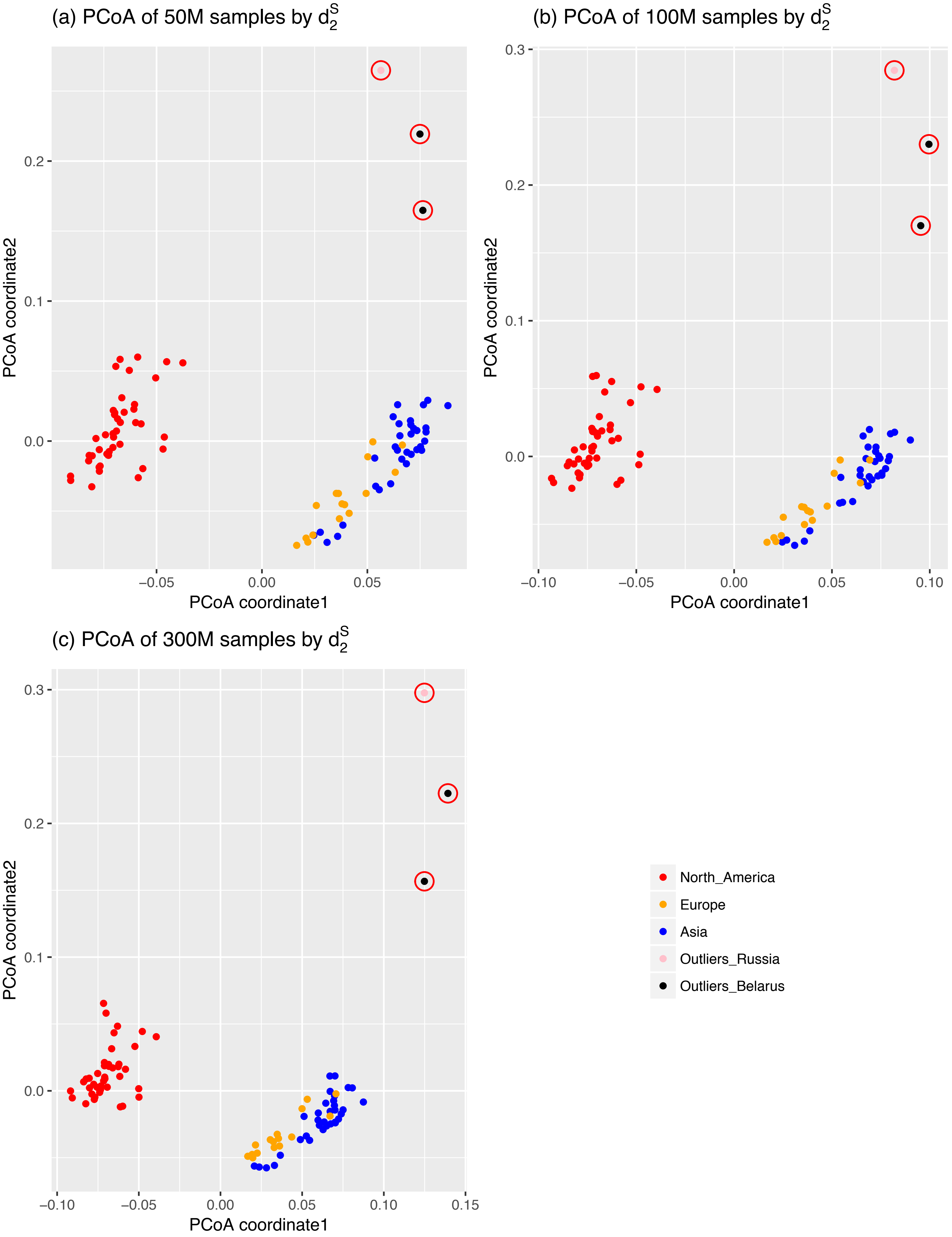
(a) PCoA of 50M samples by d_2^S
(b) PCoA of 100M samples by d_2^S
(c) PCoA of 300M samples by d_2^S
PCoA coordinate1
PCoA coordinate2
North_America
Europe
Asia
Outliers_Russia
Outliers_Belarus

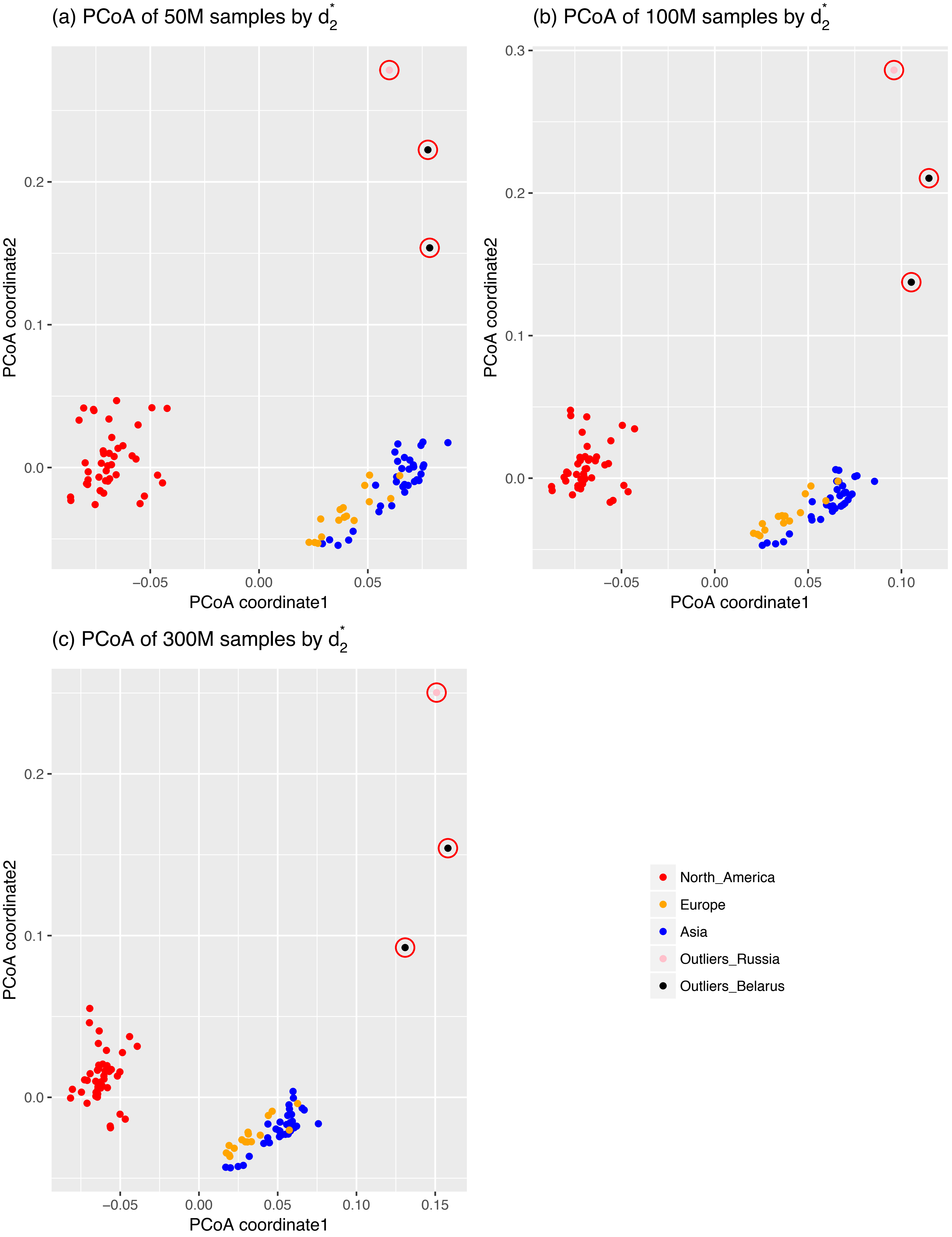

(a) PCoA of 50M samples by d_2^*
(b) PCoA of 100M samples by d_2^*
(c) PCoA of 300M samples by d_2^*
PCoA coordinate1
PCoA coordinate2
North_America
Europe
Asia
Outliers_Russia
Outliers_Belarus
