# Supplementary material for: Alignment-free genome comparison enables accurate geographic sourcing of white oak DNA
Source: BMC Genomics. 2018 Dec 10;19:896. doi: 10.1186/s12864-018-5253-1 (PMC6288960; doi:10.1186/s12864-018-5253-1)
Supplement: Supplementary file 6 — Details on the definitions of six alignment-free distance/dissimilarity measures between two genomes based on NGS data. (PDF 123 kb) [file 12864_2018_5253_MOESM6_ESM.pdf]

Details on the definitions of six alignment-free distance/dissimilarity measures between two genomes based on NGS data

Given two NGS data sets i and j from different samples and a given word length k, we first count the number of occurrences of all k-mers in all reads of sample i and sample j, respectively. The full set of k-mers of length k is defined as $\mathcal{A}^k$ where $\mathcal{A} = (A, T, C, G)$ for nucleotide sequences. For a given k-mer w, its number of occurrences in data set i is defined as $N_w^{(i)}$ and the frequency or the relative abundance of this k-mer is defined as $f_w^{(i)} = \frac{N_w^{(i)}}{\sum_w N_w^{(i)}}$.

In this study, we consider six distance/dissimilarity measures between two samples based on NGS data. These include the traditional Manhattan, Euclidean, and d_2 [1] distances between the frequencies of the word patterns.

The Manhattan distance (Ma) is defined as:

$$Ma = \sum_{w \in \mathcal{A}^k} |f_w^{(i)} - f_w^{(j)}|.$$

The Euclidean distance (Eu) is defined as:

$$Eu = \sqrt{\sum_{w \in \mathcal{A}^k} (|f_w^{(i)} - f_w^{(j)}|)^2}.$$

The d_2 distance is defined as:

$$d_2 = \frac{1}{2}\left(1 - \frac{\sum_{w \in \mathcal{A}^k} f_w^{(i)} f_w^{(j)}}{\sqrt{\sum_{w \in \mathcal{A}^k} (f_w^{(i)})^2}\sqrt{\sum_{w \in \mathcal{A}^k} (f_w^{(j)})^2}}\right).$$

We also investigate three recently developed background adjusted dissimilarity measures including CVTree [2], d_2^* and d_2^s [3, 4, 5, 6]. We model the background DNA sequence of a sample using m-th order Markov chain where the order m is estimated using the method developed for NGS short read data [4]. The expected number of occurrences of word w, $\mathbb{E}N_w^{(i)}$, can be calculated from the stationary probability of the first m-mer $w[1:m]$ and the transition probabilities from the n-th m-mer $w[n:n+m-1]$ to the $(n+m)$-th nucleotide $w[n+m]$:

$$\mathbb{E}N_w^{(i)} \approx L^{(i)} \mu(w[1:m]) \prod_{n=1}^{k-m} \pi(w[n:n+m-1], w[n+m])$$

where $L^{(i)}$ equals to the sum of the lengths of all reads in the i-th data set minus $(m-1)R$ where R is the total number of reads, μ is the stationary probability distribution, and π is the transition probability distribution that can be estimated

from the data. The difference between the number of occurrences of k-mer w and its expected number of occurrences is defined as $\tilde{N}_w^{(i)} = N_w^{(i)} - \mathbb{E}N_w^{(i)}$ that we refer to as the background adjusted k-mer counts. The CVTree, d_2^* and d_2^s dissimilarity measures are defined as follows.

The $CVTree$ dissimilarity is defined as:

$$CVTree = \frac{1}{2}\left(1 - \frac{\sum_{w\in\mathcal{A}^k} \hat{f}_w^{(i)} \hat{f}_w^{(j)}}{\sqrt{\sum_{w\in\mathcal{A}^k} (\hat{f}_w^{(i)})^2}\sqrt{\sum_{w\in\mathcal{A}^k} (\hat{f}_w^{(j)})^2}}\right),$$

where $\hat{f}_w^{(i)} = \frac{\tilde{N}_w^{(i)}}{\mathbb{E}N_w^{(i)}}$. $CVTree$ calculates $\mathbb{E}N_w^{(i)}$ by assuming a $(k-2)$-th order Markov chain for genomic sequences.

The d_2^* dissimilarity is defined as:

$$d_2^* = \frac{1}{2}\left(1 - \frac{\sum_{w\in\mathcal{A}^k} \bar{f}_w^{(i)} \bar{f}_w^{(j)}}{\sqrt{\sum_{w\in\mathcal{A}^k} (\bar{f}_w^{(i)})^2}\sqrt{\sum_{w\in\mathcal{A}^k} (\bar{f}_w^{(j)})^2}}\right),$$

where $\bar{f}_w^{(i)} = \frac{\tilde{N}_w^{(i)}}{\sqrt{\mathbb{E}N_w^{(i)}}}$.

The d_2^s dissimilarity is defined as:

$$d_2^s = \frac{1}{2}\left(1 - \frac{\sum_{w\in\mathcal{A}^k} \tilde{f}_w^{(i)} \tilde{f}_w^{(j)}}{\sqrt{\sum_{w\in\mathcal{A}^k} (\tilde{f}_w^{(i)})^2}\sqrt{\sum_{w\in\mathcal{A}^k} (\tilde{f}_w^{(j)})^2}}\right),$$

where $\tilde{f}_w^{(i)} = \frac{\tilde{N}_w^{(i)}}{((\tilde{N}_w^{(i)})^2+(\tilde{N}_w^{(j)})^2)^{\frac{1}{4}}}$ and $\tilde{f}_w^{(j)} = \frac{\tilde{N}_w^{(j)}}{((\tilde{N}_w^{(i)})^2+(\tilde{N}_w^{(j)})^2)^{\frac{1}{4}}}$.

References

[1] Torney DC, Burks C, Davison D, Sirotkin KM. Computation of d2: a measure of sequence dissimilarity. In: Computers and DNA: the proceedings of the Interface between Computation Science and Nucleic Acid Sequencing Workshop, held December 12 to 16, 1988 in Santa Fe, New Mexico/edited by George I. Bell, Thomas G. Marr. Redwood City, Calif.: Addison-Wesley Pub. Co., 1990.; 1990. .

[2] Qi J, Luo H, Hao B. CVTree: a phylogenetic tree reconstruction tool based on whole genomes. Nucleic Acids Research. 2004;32(suppl_2):W45–W47.

[3] Song K, Ren J, Zhai Z, Liu X, Deng M, Sun F. Alignment-free sequence comparison based on next-generation sequencing reads. Journal of computational biology. 2013;20(2):64–79.

[4] Ren J, Song K, Deng M, Reinert G, Cannon CH, Sun F. Inference of Markovian properties of molecular sequences from NGS data and applications to comparative genomics. Bioinformatics. 2015;32(7):993–1000.

[5] Wan L, Reinert G, Sun F, Waterman MS. Alignment-free sequence comparison (II): theoretical power of comparison statistics. Journal of Computational Biology. 2010;17(11):1467–1490.

[6] Reinert G, Chew D, Sun F, Waterman MS. Alignment-free sequence comparison (I): statistics and power. Journal of Computational Biology. 2009;16(12):1615–1634.
