# Supplementary material for: Alignment-free genome comparison enables accurate geographic sourcing of white oak DNA
Source: BMC Genomics. 2018 Dec 10;19:896. doi: 10.1186/s12864-018-5253-1 (PMC6288960; doi:10.1186/s12864-018-5253-1)
Supplement: Supplementary file 7 — Table S1. Library construction, sequencing, and genome enrichment methods used for all DNA libraries in this study. (PDF 29 kb) [file 12864_2018_5253_MOESM7_ESM.pdf]

Study	# of samples	Sequence Platform	Genomic Library Preparation	Read length (bp)	NCBI Bioproject or SRA accessions
White oak reference dataset	92	Illumina HiSeq2000	Tru-Seq, total genomic DNA	Single-end, 101bp	BioProject PRJNA269970
California Valley white oak	9	Illumina HiSeq2500	Nextera long-insert Mate-pair, total genomic DNA	Paired-end, 250 bp	BioProject PRJNA308314
	2	Illumina HiSeq2500	Nextera Mate-pair short-insert (550 bp), one PCR-free, one PCR-enriched, total genomic DNA	Paired-end, 150 bp per end	BioProject PRJNA308314
Swiss Pedunculate white oak	8	Illumina HiSeq2000	Nextera Mate-pair short-insert (400 bp), total genomic DNA	Paired-end, 100 bp	BioProject PRJNA327502
	22	PacBio-SMRT	Nextera Mate-pair long-insert (3,000 bp), total genomic DNA	Single-end, 2,489—7,622 bp	BioProject PRJNA327502
RAD-Seq data from white oaks	5	Illumina HiSeq2500	RAD-Seq with PstI selection; samples independent from the reference trees	Single-end, 91 bp	Q. bicolor, SRR5632514 Q. stellata, SRR5632513 Q. lobata, SRR5632586 Q. robur, SRR5632600 Q. dentata, SRR5632587
	2	Illumina HiSeq2500	RAD-Seq with PstI selection; samples identical to two reference trees	Single-end, 91 bp	Q. mongolica, SRR5284345 Q. petraea, SRR5284338
